# Supplementary material for: Estrogen promotes innate immune evasion of Candida albicans through inactivation of the alternative complement system
Source: Cell Rep. 2022 Jan 4;38(1):110183. doi: 10.1016/j.celrep.2021.110183 (PMC8755443; doi:10.1016/j.celrep.2021.110183)
Supplement: Document S1. Figures S1 and S2 and Tables S2 — –S7 [file mmc1.pdf]

Cell Reports, Volume *38*

Supplemental information

Estrogen promotes innate immune evasion of *Candida albicans* through inactivation of the alternative complement system

Pizga Kumwenda, Fabien Cottier, Alexandra C. Hendry, Davey Kneafsey, Ben Keevan, Hannah Gallagher, Hung-Ji Tsai, and Rebecca A. Hall

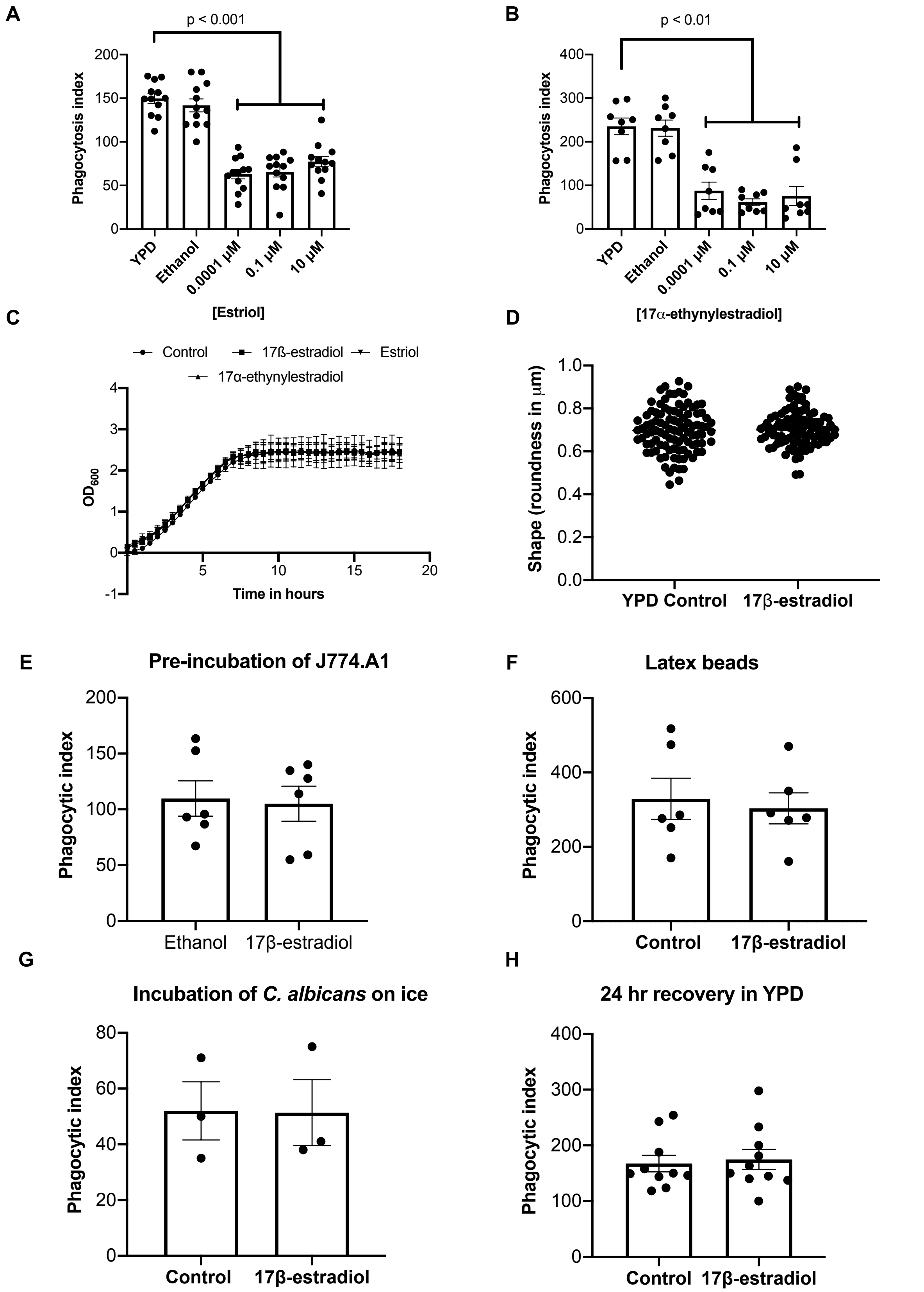

Figure S1. Oestrogen inhibits *C. albicans* phagocytosis, but does not affect fungal growth or morphology related to Figure 1. A) J774A.1 phagocytosis rates of *C. albicans* (SC5314) grown in the presence of estriol.

B) J774A.1 phagocytosis rates of *C. albicans* grown in the presence of 17α-ethynylestradiol. **C)** *C. albicans* cells were grown in 96-welled plate in YPD broth supplemented with 10 μM 17β-estradiol, 10 μM 17α-ethynylestradiol or 10 μM estriol. Optical densities (OD) of the cultures were recorded every 30 min for 18 h. **D)** *C. albicans* was grown in YPD broth supplemented with 17β-estradiol 10 μM for 4 h. Cells were washed with PBS, stained with concanavalin A, imaged by microscopy and analysed for shape. **E)** J774A.1 macrophages preincubated with 10 μM 17β-estradiol were infected with *C. albicans* cells grown in YPD and phagocytosis rates quantified. **F)** Latex beads were incubated at room temperature in PBS with or without 10 μM 17β-estradiol. Beads were washed and co-incubated with J774A.1 macrophages and phagocytosis rates quantified. **G)** *C. albicans* cells were incubated in PBS at 4°C for 4 h with or without 10 μM 17β-estradiol. Cells were washed and co-incubated with J774A.1 macrophages and phagocytosis rates quantified **H)** *C. albicans* cells previously grown in YPD with or without 10 μM 17β-estradiol were harvested, washed and re-incubated in fresh YPD for 24 h. Cells were co-incubated with J774A.1 macrophages and phagocytosis rates quantified. All data represent the mean ± SEM from at least three independent experiments.

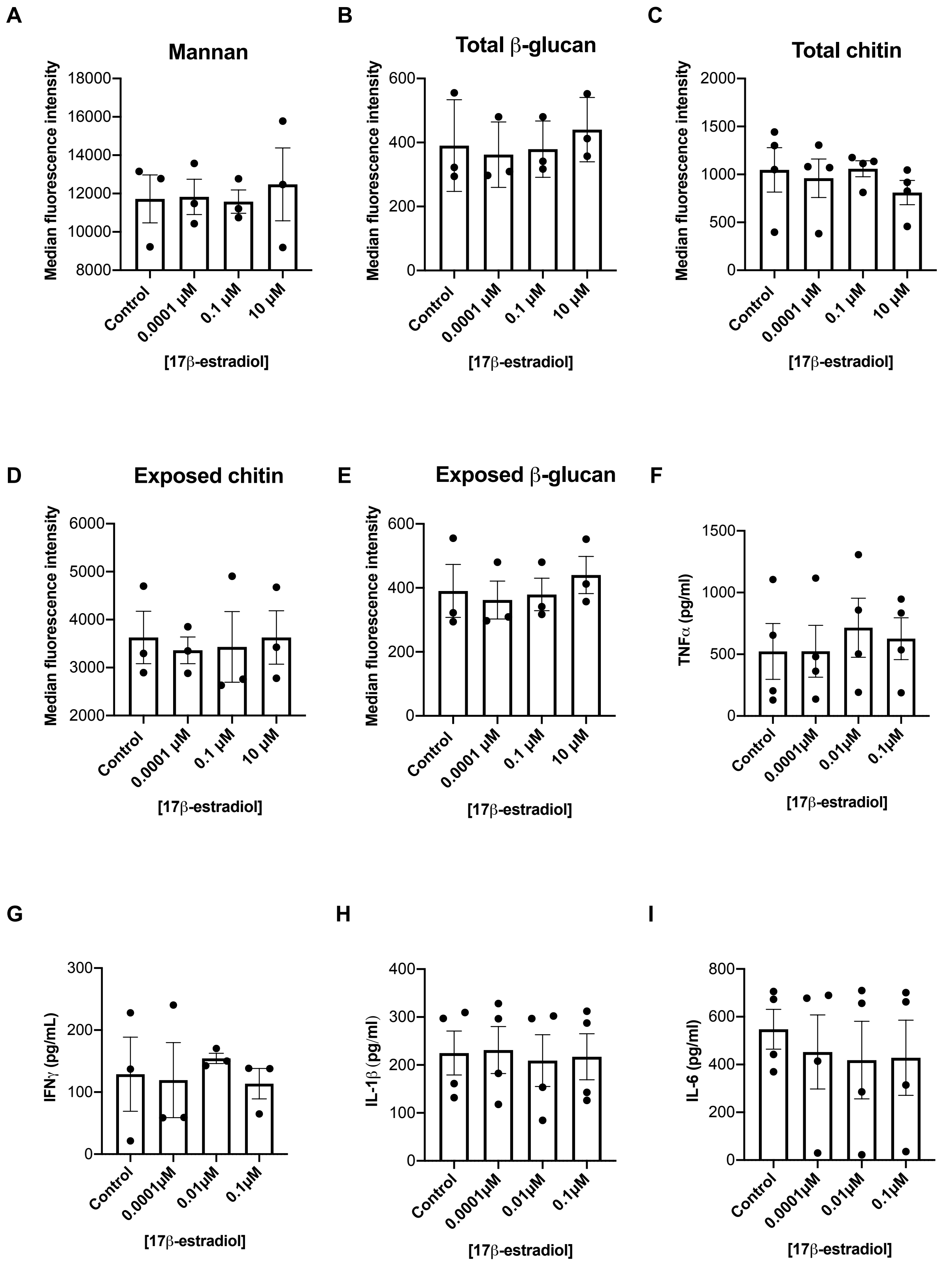

Figure S2. Reduced phagocytosis rates are not correlated with altered cytokine secretion or gross changes in cell wall carbohydrates related to Figure 2. *C. albicans* cells were grown in YPD with or without 10 μM 17β-estradiol. Cells were harvested, washed in PBS, fixed with 4% PFA and stained for **A**) total mannan **B**)

total glucan **C**) total chitin **D)** exposed chitin **E)** exposed glucan. Staining was quantified by flow cytometry and median fluorescence intensities (MFI) determined. All data represent the mean ± SEM from at least three independent biological experiments. *C. albicans* cells were grown in YPD with or without 10 μM 17β-estradiol for 4 h. Cells were washed, fixed with 4% PFA and co-incubated with PBMCs for 24h. Secretion of **F**) TNFα, **G)** IFNγ, **H)** IL-1β and **I)** IL-6 by PBMCs was quantified by ELISA. Data represent the mean ± SEM from four independent experiments using different donors.

Table S2. Gene Ontology analysis of differentially regulated genes (related to Figure 3)

GO Term	Cluster	Adjusted P-value	Genes
**Up regulated genes**			
Hormone binding	2 / 59 genes (3.4%)	0.00473	*CDR1, EBP1*
snoRNA binding	4 / 59 genes (6.8%)	0.00541	*BMS1, NOP14, HCA4, NOP10*
catalytic activity, acting on a rRNA	3 / 59 genes (5.1%)	0.01756	*DIM1, SPB1, CR_04170W_A*
rRNA methyltransferase activity	3 / 59 genes (5.1%)	0.01756	*DIM1, SPB1, CR_04170W_A*
U3 snoRNA binding	3 / 59 genes (5.1%)	0.02147	*BMS1, NOP14, HCA4*
FMN binding	3 / 59 genes (5.1%)	0.0737	*OYE32, EBP1, OYE23*
**Down regulated genes**			
Oxidoreductase activity	19 / 76 genes (25.0%)	2.07E-05	*HMX1, CSH1, IFD6, C1_04460C_A, CAT1, GCV2, C2_00180C_A, ALD5, C2_04480W_A, PST1, PST2, FOX2, POX1-3, MET13, SOU1, GRP2, HPD1, FDH1, FRE7*
Oxidoreductase activity, acting on the CH-OH group of donors, NAD or NADP as acceptor	7 / 76 genes (9.2%)	0.00078	*CSH1, IFD6, FOX2, SOU1, GRP2, HPD1, FDH1*
Oxidoreductase activity, acting on CH-OH group of donors	7 / 76 genes (9.2%)	0.00115	*CSH1, IFD6, FOX2, SOU1, GRP2, HPD1, FDH1*
Aryl-alcohol dehydrogenase (NAD+) activity	2 / 76 genes (2.6%)	0.02754	*CSH1, IFD6*
NAD(P)H dehydrogenase (quinone) activity	2 / 76 genes (2.6%)	0.05466	*PST1, PST2*
Glutaminase activity	2 / 76 genes (2.6%)	0.05466	*SNZ1, SNO1*
Glucose transmembrane transporter activity	3 / 76 genes (3.9%)	0.08953	*HGT19, HGT5, HGT10*

Table S3. Motif letter-probability matrix (related to Figure 3)

Motif	***A***	***C***	***G***	***T***

Motif 1
E-value = 1.2e-159

0.000000	0.037037	0.175926	0.787037
0.009259	0.046296	0.129630	0.814815
0.000000	0.379630	0.000000	0.620370
0.101852	0.074074	0.064815	0.759259
0.000000	0.120370	0.055556	0.824074
0.018519	0.259259	0.009259	0.712963
0.000000	0.259259	0.000000	0.740741
0.055556	0.129630	0.009259	0.805556
0.000000	0.240741	0.009259	0.750000
0.000000	0.157407	0.129630	0.712963
0.083333	0.416667	0.027778	0.472222
0.151389	0.232870	0.001389	0.614352
0.000000	0.185185	0.000000	0.814815
0.055556	0.129630	0.046296	0.768519
0.018519	0.546296	0.120370	0.314815
0.083333	0.037037	0.018519	0.861111
0.037037	0.157407	0.092593	0.712963
0.129630	0.277778	0.046296	0.546296
0.166667	0.314815	0.055556	0.462963
0.129630	0.092593	0.037037	0.740741
0.138889	0.222222	0.083333	0.555556
0.129630	0.157407	0.055556	0.657407
0.037037	0.194444	0.037037	0.731481
0.194444	0.000000	0.111111	0.694444
0.083333	0.333333	0.120370	0.462963
0.203704	0.268519	0.027778	0.500000
0.185185	0.342593	0.046296	0.425926
0.175926	0.194444	0.037037	0.592593
0.111111	0.287037	0.101852	0.500000

Motif 2
E-value = 4.9e-04

0.321429	0.392857	0.071429	0.214286
0.232143	0.214286	0.285714	0.267857
0.357143	0.375000	0.000000	0.267857
0.071429	0.464286	0.000000	0.464286
0.125000	0.428571	0.107143	0.339286
0.107143	0.178571	0.000000	0.714286
0.232143	0.464286	0.017857	0.285714
0.089286	0.392857	0.053571	0.464286
0.178571	0.678571	0.125000	0.017857
0.166964	0.466964	0.002679	0.363393
0.000000	0.821429	0.053571	0.125000
0.232143	0.375000	0.017857	0.375000
0.000000	0.660714	0.000000	0.339286
0.000000	0.732143	0.000000	0.267857
0.196429	0.482143	0.000000	0.321429
0.017857	0.660714	0.089286	0.232143
0.107143	0.589286	0.035714	0.267857
0.000000	0.589286	0.000000	0.410714
0.000000	0.821429	0.000000	0.178571
0.375000	0.089286	0.053571	0.482143
0.392857	0.482143	0.125000	0.000000

Motif 3
E-value = 2.5e-009

0.805556	0.055556	0.138889	0.000000
0.763889	0.097222	0.000000	0.138889
0.500000	0.430556	0.069444	0.000000
0.722222	0.069444	0.111111	0.097222
0.902778	0.013889	0.083333	0.000000
0.652778	0.222222	0.000000	0.125000
0.847222	0.000000	0.152778	0.000000
0.972222	0.027778	0.000000	0.000000
0.847222	0.083333	0.069444	0.000000
0.902778	0.027778	0.000000	0.069444
1.000000	0.000000	0.000000	0.000000
0.791667	0.208333	0.000000	0.000000
1.000000	0.000000	0.000000	0.000000
1.000000	0.000000	0.000000	0.000000

Table S4. Transcription factors that bind identified DNA binding motifs (related to Figure 3)

**Motif**	***S. cerevisiae***	***C. albicans***	**Description on CGD**
**Motif 1**	Azf1	CR_02510W	Induced by Mnl1 under weak acid stress
	Fkh1	Fkh2	Morphogenesis regulator
	Hsf1	Cta8	Mediates heat shock transcriptional induction
	Sfl1	Sfl1	Negative regulation of morphogenesis, flocculation and virulence
	Ste12	Cph1	Mating, and filamentation on solid media repressed
	Cup2	NA	
	Fkh2	NA	
**Motif 2**	Haa1	Cup2	Required for normal resistance to copper
	Msn2	Msn4	Similar to *S. cerevisiae* Msn4
	Msn4	Msn4	Similar to *S. cerevisiae* Msn4
	Ygr067c	Try5	Regulator of yeast form adherence
	Rap1	Rap1	Binds telomeres and regulatory sequences in DNA
	Yml081w	Zms1	Spider biofilm induced
	Cha4	Tea1	Putative transcription factor with zinc cluster DNA-binding motif
	Nrg1	Nrg1	Regulates chlamydospore formation, hyphal gene induction, virulence
	Rgm1	NA	
	Rph1	Rph1	Ortholog(s) have DNA-binding transcription repressor activity
	Gis1	NA	
	Usv1	Bcr1	Regulates a/alpha biofilm formation, matrix, cell-surface-associated genes
	Yer130c	Mnl1	induces transcripts of stress response genes via SLE (STRE-like) elements
	Ypr022c	C3_06150W	Ortholog(s) have role in negative regulation of transcription by RNA polymerase II
**Motif 3**	Azf1	CR_02510W	Induced by Mnl1 under weak acid stress
	Cup2	NA	
	Hcm1	Hcm1	Similar to *S. cerevisiae* Hcm1

	Fkh2	NA	

Table S5. GO Ter analysis of *BCR1* regulated gene (related to Figure 3)

GO ID	GO Term	% of input genes	p-value	Genes
GO:0009986	cell surface	15.56%	4.91E-07	*ALS4, ECM331, GPD2, PGA13, RBT5, SAP10, SIM1*
GO:0005886	plasma membrane	15.56%	4.61E-05	*AQY1, CFL5, DIP5, ECM331, GPR1, JEN1, RBT5*
GO:0009277	fungal-type cell wall	13.33%	6.29E-08	*ALS4, ECM331, PGA13, RBT5, SAP10, SIM1*
GO:0016021	integral component of membrane	13.33%	9.06E-05	C1_00660C_A, *GPR1, JEN1, MAL31, RBD1, UFE1*
GO:0005634	nucleus	13.33%	1.81E-03	CR_03700C_A, *GRF10, SEF2, SFL2, WOR2, WOR3*

Table S6. Strains used in this study (related to STAR methods)

Strain	Genotype	Source/reference
SC5314	Wild type	(Gillum *et al.* 1984)
SVS006B	Clinical isolate	Prof Ramage, Glasgow University
SVS062A	Clinical isolate	Prof Ramage, Glasgow University
SN152	*arg4Δ/arg4Δ leu2Δ/leu2Δ his1Δ/his1Δ URA3/ura3Δ:: λimm434 IRO1/iro1Δ:: λimm434*	(Noble *et al.* 2010)
SN250	*his1Δ/his1Δ, leu2Δ::C. dubliniensis HIS1 /leu2Δ::C. maltosa LEU2-arg4Δ/arg4Δ, URA3/ura3Δ::imm434-IRO1/iro1Δ::imm434*	(Noble et al. 2010)
SN250-CIP30	As SN250 but *RPS1/rps1::*CIP30	This study
*rob1*Δ	As SN152 but *rob1Δ::C. dubliniensis HIS1/rob1Δ::C. maltose LEU2*	(Noble *et al.* 2010)

*bcr1*Δ	*arg4Δ/arg4Δ leu2Δ/leu2Δ his1Δ/his1Δ URA3/ura3Δ::λimm434 IRO1/iro1Δ:: λimm434 bcr1::LEU/bcr1::HIS1*	(Noble *et al.* 2010)
*bcr1*Δ-CIP30	As *bcr1*Δ but with *RPS1/rps1*::CIP30	This study
*bcr1*Δ-CIP30-*BCR1*	As *bcr1*Δ but with *RPS1/rps1*::CIP30-*BCR1*	This study
*bcr1*Δ-CIP30	As *bcr1*Δ but with *RPS1/rps1*::CIP30-*pTEF2-GPD2*	This study
*gpd2*Δ	As SN152 but *gpd2Δ::C. dubliniensisHIS1/gpd2Δ::C. maltose LEU2*	(Noble *et al.* 2010)
*gpd2*Δ–CIP30	As SN152 but *gpd2Δ::C. dubliniensisHIS1/gpd2Δ::C. maltose LEU2, RPS1/rps1::CIP30*	This study
*gpd2*Δ-CIP30-*GPD2*	As SN152 but *gpd2Δ::C. dubliniensisHIS1/gpd2Δ::C. maltose LEU2RPS1/rps1::CIP30-GPD2*	This study
CAI4	*ura3::imm434/ura3::imm434 iro1/iro1::imm434*	(Fonzi and Irwin 1993)
CAI4-pSM2	*ura3::imm434/ura3::imm434 iro1/iro1::imm434::*pSM2	(Hall *et al.* 2010)
CAI4-pSM2-*GPD2*	*ura3::imm434/ura3::imm434 iro1/iro1::imm434::*pSM2-p*TEF2-GPD2*	This study
*ebp1*Δ	As CAI4 but *ebp1::*dlp200/*ebp1::*dlp200	This study
*ebp1*Δ-pSM2	As CAI4 but *ebp1::*dlp200/*ebp1*::dlp200-pSM2	This study
*ebp1*Δ-pSM2-*EBP1*	As *ebp1*Δ but with pSM2-*EBP1*	This study
*ebp1*Δ-pSM2-*GPD2*	As *ebp1*Δ but *with* pSM2-p*TEF2-GPD2*	This study
CAF2-1	*URA3/ura3:: λimm434*	(Fonzi and Irwin 1993)
*cdr1*Δ	As CAF2-1, but *crd1::hisG/cdr1:hisG-URA3-HisG*	(Sanglard *et al.* 1997)
*cdr1/2*Δ	As CAF2-1, but *crd1::hisg/cdr1:hisG, cdr2::hisG-URA3-hisG/cdr2::hisG*	(Sanglard *et al.* 1997)

Table S7. Primers used in this study (related to STAR methods)

**Primer**	**Sequence**
GPD2-SacI-F	CTCCGAGCTCGGTGATGGTGATGGTGATGG
GPD2-NotI-R	GGAGAGCGGCCGCTGGTAAATTGGACAACGAGTGG
GPD2-OE-F	**GGAGAG**ccgcggATGACTACTTCCCCATATCCAAT
GPD2-OE-R	**GGAGAG**gcggccgcACGCAGAGAACAAGAACGTC
EBP1-5F:	CTCCGATATCATCGCATGAG
EBP1-5R:	GGAGAGGAGCTCctgatgatatgataatttgc
EBP1-3F:	GGAGAGAAGCTTGGGAATGAAGTTCTATTAGC
EBP1-3R:	GGAGAGGGTACCgttacatctactactacagg
EBP1-CF	ACAACAACAAGAAGACACGG
EBP1-CR	GAATCTATGGTCAAGTAGAAG
URA3-F	GCCTCACCAGTAGCACAGCGATTA
EBP1-F	GGAGAGccgcggGAAACGCCTTGTTGGACAAA
EBP1-R	GGAGAGgcggccgcTGATAATAACCAGACCACCACA
BCR1-F	GGAGAGgagctcCCAACCAACATTCCAATTCC
BCR1-R	GGAGAGgcggccgcAACCTTTACCTTTGGATTTTTGA
RT-ACT1-F	CCTACGTGTACTTGTGCAAGGCAA
RT-ACT1-R	TAGTTGTGTGCACTGAGCGTCGAA
RT-GPD2-F	GCCAACGAAGTTGCCAAAGGT
RT-GPD2-R	AGGCACCAGCAATAGAGGCA
RT-EBP1-F	TGCGCCATCAGCAGTTTATTGG
RT-EBP1-R	TCCAACAAGTAACCATGAGCACCA
