# Supplementary material for: Estrogen promotes innate immune evasion of Candida albicans through inactivation of the alternative complement system
Source: Cell Rep. 2022 Jan 4;38(1):110183. doi: 10.1016/j.celrep.2021.110183 (PMC8755443; doi:10.1016/j.celrep.2021.110183)
Supplement: Document S2. Article plus supplemental information [file mmc3.pdf]

Cell Reports

Article

Estrogen promotes innate immune evasion of *Candida albicans* through inactivation of the alternative complement system

Graphical abstract

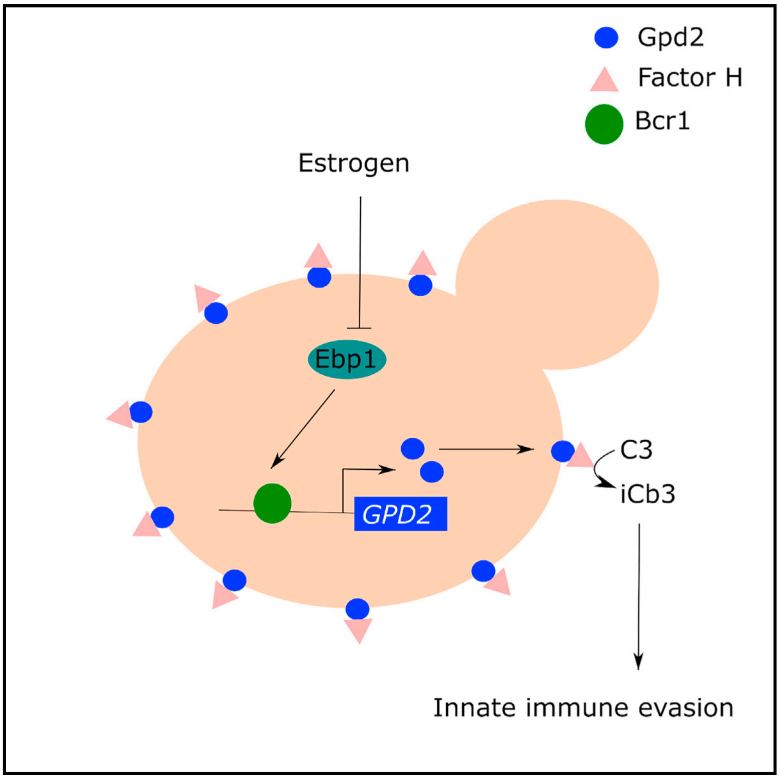

Highlights

- Estrogen promotes the virulence of *Candida albicans* by reducing phagocytosis
- Estrogen-adapted *C. albicans* binds more Factor H on its cell surface
- Estrogen-induced innate immune evasion of *C. albicans* is dependent on Gpd2
- Overexpression of *GPD2* promotes the virulence of *C. albicans*

Authors

Pizga Kumwenda, Fabien Cottier, Alexandra C. Hendry, ..., Hannah Gallagher, Hung-Ji Tsai, Rebecca A. Hall

Correspondence

r.a.hall@kent.ac.uk

In brief

Women with high estrogen levels have an increased risk of developing genital thrush caused by *Candida albicans*. Kumwenda et al. show that *C. albicans* grown in estrogen is not recognized and killed efficiently by white blood cells. This reduced immune recognition could enable *C. albicans* to proliferate and cause infection.

Kumwenda et al., 2022, Cell Reports *38*, 110183
January 4, 2022 © 2021 The Authors.
https://doi.org/10.1016/j.celrep.2021.110183

CellPress

Cell Reports

CellPress
OPEN ACCESS

Article

Estrogen promotes innate immune evasion of *Candida albicans* through inactivation of the alternative complement system

Pizga Kumwenda,[1] Fabien Cottier,[1] Alexandra C. Hendry,[2] Davey Kneafsey,[2] Ben Keevan,[1] Hannah Gallagher,[1] Hung-Ji Tsai,[1] and Rebecca A. Hall[1,2,3,*]

[1]Institute of Microbiology and Infection, School of Biosciences, University of Birmingham, Birmingham B15 2TT, UK
[2]Kent Fungal Group, Division of Natural Sciences, School of Biosciences, University of Kent, Canterbury CT2 7NJ, UK
[3]Lead contact
*Correspondence: r.a.hall@kent.ac.uk
https://doi.org/10.1016/j.celrep.2021.110183

SUMMARY

Candida albicans is a commensal of the urogenital tract and the predominant cause of vulvovaginal candidiasis (VVC). Factors that increase circulatory estrogen levels such as pregnancy, the use of oral contraceptives, and hormone replacement therapy predispose women to VVC, but the reasons for this are largely unknown. Here, we investigate how adaptation of *C. albicans* to estrogen impacts the fungal host-pathogen interaction. Estrogen promotes fungal virulence by enabling *C. albicans* to avoid the actions of the innate immune system. Estrogen-induced innate immune evasion is mediated via inhibition of opsonophagocytosis through enhanced acquisition of the human complement regulatory protein, Factor H, on the fungal cell surface. Estrogen-induced accumulation of Factor H is dependent on the fungal cell surface protein Gpd2. The discovery of this hormone-sensing pathway might pave the way in explaining gender biases associated with fungal infections and may provide an alternative approach to improving women's health.

INTRODUCTION

Microbial infections exhibiting gender bias are common. This bias may predispose one sex to infection over the other, or result in one sex exhibiting severer infection outcomes (Ghazeeri et al., 2011; García-Gómez et al., 2013; van Lunzen and Altfeld, 2014). Sex hormones such as estrogen, testosterone, and progesterone regulate many functions of the immune system, and generally males are more prone to infection than females, as overall immune responses are lower in the male population (Klein, 2000; McClelland and Smith, 2011; Taneja, 2018). Besides the impact of sex hormones on immune cell function, sex hormones also have a direct effect on microbial pathogenicity by increasing microbial persistence, metabolism, and virulence gene expression (Amirshahi et al., 2011; Chotirmall et al., 2012).

The opportunistic fungal pathogen *Candida albicans* is the predominant fungal colonizer of the female reproductive tract and the major cause of genital thrush (vulvovaginal candidiasis [VVC]). Approximately 75% of the female population will encounter at least one episode of VVC in their lifetime, while up to 15% experience recurrent infection (RVVC) defined as four or more episodes in a 12-month period (Sobel, 1997). Although not life threatening, mucosal infections are expensive to treat, impact the quality of life, and increase population morbidity.

One of the major risk factors associated with the development of VVC is elevated levels of estrogen which occur as a result of pregnancy, the use of high-estrogen-containing oral contraceptives, and hormone replacement therapy (Sobel, 1988; Dennerstein and Ellis, 2001). Therefore, estrogen plays a key role in predisposing women to VVC, but the precise mechanisms for this are unknown. Estrogen promotes glycogen production at the vaginal mucosa, providing a nutrient rich environment for the expansion of *C. albicans* (Dennerstein and Ellis, 2001). In mouse models of VVC, were pseudoestrus is induced to maintain fungal colonization, vaginal epithelial cells have a diminished ability to control the growth of *C. albicans* (Fidel et al., 2000). In addition, in mice, estrogen decreases the infiltration of phagocytes into the vaginal cavity and suppresses cell-mediated immunity (Salinas-Muñoz et al., 2018). However, in rats, preincubation of *C. albicans* with estrogen prior to vaginal infection enhances fungal survival (Kinsman and Collard, 1986), suggesting that in addition to affecting host immunity, estrogen may directly affect the virulence of *C. albicans*. In agreement with this, estrogen has been shown to promote hyphal morphogenesis of *C. albicans* (White and Larsen, 1997), a key virulence factor of the fungus. Furthermore, an estrogen-binding protein (Ebp1) has been identified in *C. albicans* (Madani et al., 1994), although the importance of this protein in VVC is not known. Therefore, how *C. albicans* adapts to estrogen is still unclear.

The *C. albicans* cell wall is a multilayered structure consisting of an inner layer of chitin and β-glucan and an outer layer of heavily glycosylated proteins (Netea et al., 2008). The fungal cell wall is a highly dynamic structure providing rigidity, strength, and protection from the environment. In addition, many components of the cell wall act as pathogen-associated molecular

This is an open access article under the CC BY license (http://creativecommons.org/licenses/by/4.0/).

patterns (PAMPs) and are recognized by the innate immune system (Netea et al., 2008; Hall and Gow, 2013). Recently, remodeling of the cell wall in response to adaptation to host environments has been shown to regulate the host-pathogen interaction (Wheeler and Fink, 2006; Wheeler et al., 2008; Hall, 2015; Ballou et al., 2016; Hopke et al., 2016; Sherrington et al., 2017; Lopes et al., 2018; Pericolini et al., 2018; Pradhan et al., 2018, 2019; Tripathi et al., 2020; Williams and Lorenz, 2020). In addition to direct recognition of cell wall PAMPs mediating phagocytosis, *C. albicans* also activates the alternative complement system, resulting in the deposition of complement proteins (i.e., C3) on the fungal cell surface, resulting in opsonophagocytosis (Kozel et al., 1996). However, *C. albicans* can evade opsonophagocytosis through the binding of complement regulatory proteins to its cell surface, which inactivate the complement cascade (Meri et al., 2002). Here we show that *C. albicans* does adapt to estrogen and that this adaptation perturbs the host-pathogen interaction, inhibiting phagocytosis of the fungal pathogen. Avoidance of the innate immune system was mediated via the fungal cell surface protein, Gpd2, recruiting the human complement regulatory protein, Factor H, to the fungal cell surface, inactivating the alternative complement system.

RESULTS

Adaptation of *C. albicans* to estrogen promotes innate immune evasion

Pregnant women and women taking high-estrogen-containing oral contraceptives are more prone to VVC (Gonçalves et al., 2016), while VVC occurs less frequently in postmenopausal women, indicating that estrogen may play a role in promoting the virulence of *C. albicans*. There are four main forms of estrogen: estrone (E1), 17β-estradiol (E2), estriol (E3), and 17α-ethynylestradiol (EE2). Estrone is the weakest form of estrogen produced by the ovaries and adipose tissue and is only found in menopausal women, while 17β-estradiol is the strongest form of estrogen produced by the ovaries and has been associated with many gynecological disorders. Estriol is a by-product from the metabolism of estradiol, and as such is found in high concentrations during pregnancy. Finally, 17α-ethynylestradiol is a synthetic estrogen used in oral contraceptive pills. To ascertain whether adaptation of *C. albicans* to estrogen affects the host-pathogen interaction, we grew *C. albicans* cells in the presence of physiological (0.0001 μM) and super-physiological (0.1 μM, 10 μM) concentrations of 17β-estradiol, estriol, or 17α-ethynylestradiol, and quantified the phagocytosis rates. Physiological and super-physiological concentrations of all three forms of estrogen significantly inhibited macrophage and neutrophil phagocytosis of *C. albicans*, resulting in a 50% drop in phagocytosis compared with the ethanol vehicle control (Figures 1A–1C,S1A, and S1B). The reduced rates of *C. albicans* phagocytosis were independent of any impact of estrogen on fungal growth (Figure S1C) or morphology (Figure S1D). Given that all tested forms of estrogen elicited similar results, all subsequent experiments were performed only with 17β-estradiol.

To determine whether the observed inhibition of phagocytosis is truly mediated by adaptation of *C. albicans* to estrogen, and not a result of residual estrogen coating the surface of *C. albicans* inhibiting phagocyte function, we pretreated macrophages with estrogen prior to the addition of *C. albicans* or latex beads. Macrophages preincubated with estrogen phagocytosed *C. albicans* at rates comparable with those in non-treated cells (Figure S1E), suggesting that under the tested conditions estrogen does not directly impact the ability of macrophages to phagocytose target particles. Preincubating inert particles such as latex beads with estrogen had no effect on the ability of macrophages to phagocytose the particles (Figure S1F), suggesting that any residual estrogen on the surface of particles does not interfere with the ability of macrophages to phagocytose them. In agreement with this, incubation of *C. albicans* with estrogen on ice, which inhibits fungal growth and metabolism, did not affect phagocytosis rates (Figure S1G). Furthermore, reinoculation of estrogen-adapted *C. albicans* cells into fresh YPD medium restored *C. albicans* phagocytosis rates (Figure S1H). Therefore, in response to estrogen, *C. albicans* undergoes some form of adaptation that alters the host-pathogen interaction.

Finally, we tested several vaginal *C. albicans* isolates and observed that all isolates displayed reduced phagocytosis after adaptation to estrogen, confirming that estrogen-induced immune evasion is a general trait of clinically relevant *C. albicans* (Figure 1D). Taking these data together, we conclude that the reduction in *C. albicans* phagocytosis is due to the fungus reversibly adapting to the estrogen, and that this adaptation interferes with the host mechanism of fungal clearance.

Hormone-induced innate immune evasion is specific to estrogen

Progesterone has been shown to affect phagocytosis rates (György, 2017) and is structurally similar to estrogen. Therefore, we hypothesized that *C. albicans* may adapt to progesterone in a similar way to estrogen to evade the innate immune system. *C. albicans* cells pre-exposed to physiological and super-physiological concentrations of progesterone exhibited phagocytosis rates comparable with those of *C. albicans* cells grown in YPD (Figure 1E), while *C. albicans* treated with both estrogen and progesterone still exhibited innate immune evasion (Figure 1F). Therefore, the promotion of innate immune evasion appears to be a specific and dominant attribute of estrogen.

Estrogen-induced innate immune evasion is mediated via inhibition of opsonophagocytosis

The interaction between *C. albicans* and innate immune cells can be mediated via direct detection of fungal cell wall carbohydrates and opsonin-mediated phagocytosis (opsonophagocytosis). Adaptation of *C. albicans* to a variety of environmental conditions influences the *Candida* host-pathogen interaction through altered Dectin-1 dependent recognition of β-glucan, which correlates with altered proinflammatory cytokine secretion (Wheeler and Fink, 2006; Wheeler et al., 2008; Hall, 2015; Ballou et al., 2016; Hopke et al., 2016; Sherrington et al., 2017; Lopes et al., 2018; Pericolini et al., 2018; Pradhan et al., 2018, 2019; Tripathi et al., 2020; Williams and Lorenz, 2020). However, estrogen did not affect the amount or exposure of the main cell wall carbohydrates (Figures S2A–S2E), and did not have significant impact on the section of proinflammatory cytokines (Figures S2F–S2I).

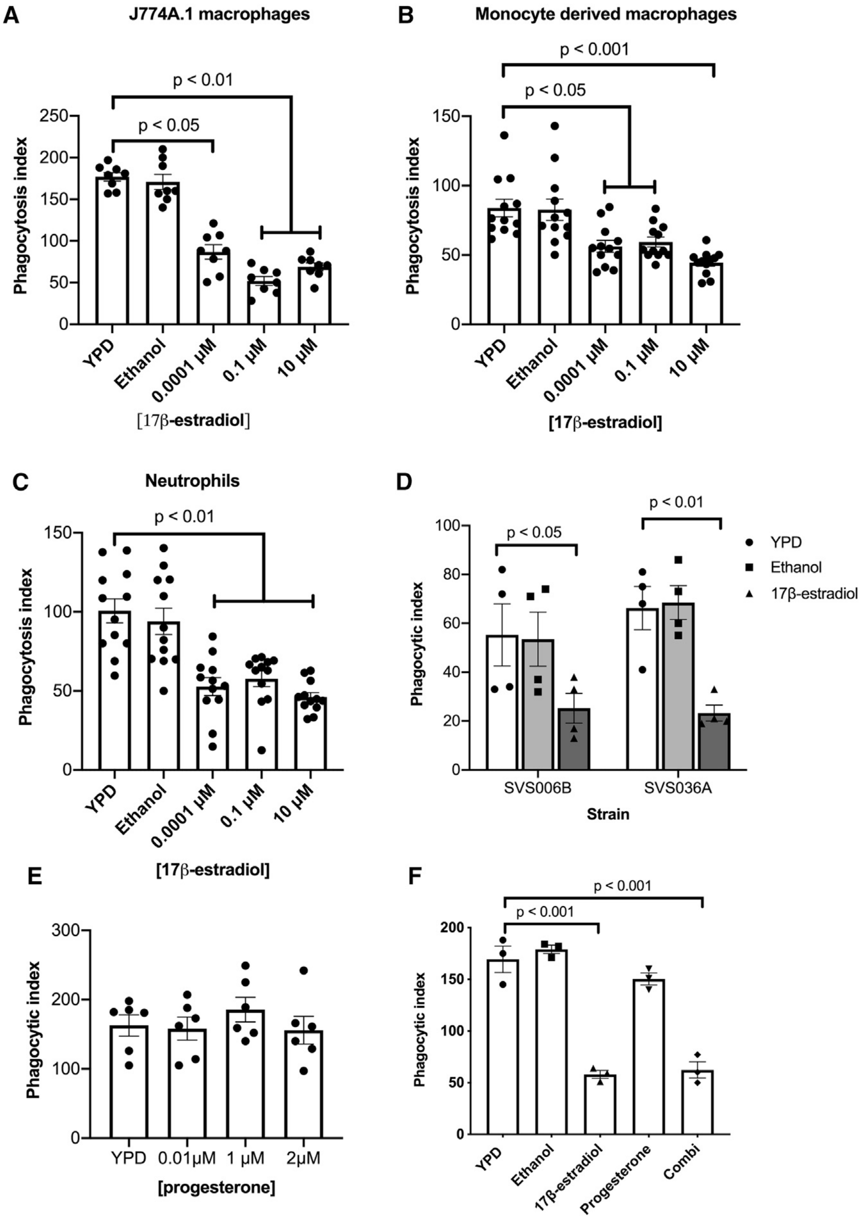

Figure 1. Estrogen promotes innate immune evasion of *C. albicans*

(A–C) *C. albicans* cells were grown in YPD with or without estrogen supplementation. Cells were harvested, washed in PBS and co-incubated with (A) J774A.1 cells, (B) human macrophages, and (C) human neutrophils.

(D) J774A.1 phagocytosis rates of *C. albicans* clinical isolates after exposure to 10 μM 17-estradiol.

(E) J774A.1 phagocytosis rates of *C. albicans* after exposure to progesterone.

(F) J774A.1 phagocytosis rates of *C. albicans* after exposure to 1 μM progesterone and 10 μM 1-estradiol, either individually or in combination (Combi).

Cells were fixed with 4% paraformaldehyde (PFA), imaged by microscopy, scored using ImageJ software, and the phagocytic index determined. All data represent the mean ± SEM from at least three independent biological experiments. Individual data points represent each independent biological replicate. See also Figure S1.

In addition to direct detection of cell wall PAMPs, *C. albicans* activates the alternative complement cascade, resulting in the deposition of complement (C3) on its surface inducing opsonophagocytosis via the complement receptors CR1 and CR3. To investigate whether estrogen-induced innate immune evasion was mediated via inhibition of complement, we assessed phagocytosis rates in heat-inactivated serum, where the major complement proteins (i.e., C3) are denatured. Overall, the phagocytosis rates of *C. albicans* in heat-inactivated serum were lower than in live serum, confirming the importance of opsonophagocytosis in the recognition of *C. albicans*. However, adaptation of *C. albicans* to estrogen did not result in further evasion of phagocytosis in heat-inactivated serum (Figure 2A), suggesting that pre-exposure of *C. albicans* to estrogen affects complement activation. Supplementation of heat-inactivated serum with purified C3 restored estrogen-induced immune evasion (Figure 2B), confirming that estrogen-induced immune evasion likely occurs through the avoidance of opsonophagocytosis.

Next, we tested whether adaptation of *C. albicans* to estrogen affected the deposition of C3 and C3b on the fungal cell surface. Estrogen-adapted cells exhibited enhanced C3 and C3b deposition compared with non-adapted cells (Figure 2C), suggesting that adaptation to estrogen promotes C3 and C3b binding on the *C. albicans* cell surface. Increased deposition of complement is normally associated with increased phagocytosis. However, processing of C3 can be inhibited by the recruitment of host regulatory proteins such as Factor H, and several pathogens are known to avoid opsonophagocytosis through enhanced recruitment of Factor H (Dasari et al., 2018; van der Maten et al., 2018). Therefore, the ability of estrogen-adapted *C. albicans* cells to bind Factor H was quantified. Estrogen-adapted cells bound significantly more Factor H compared with the solvent control (Figure 2D), suggesting that estrogen-adapted *C. albicans* evades the innate immune system through enhanced Factor H recruitment and decreased opsonophagocytosis.

Bcr1 plays a key role in estrogen-induced innate immune evasion

Altered deposition of complement on the fungal cell surface in response to estrogen stimulation suggests that estrogen

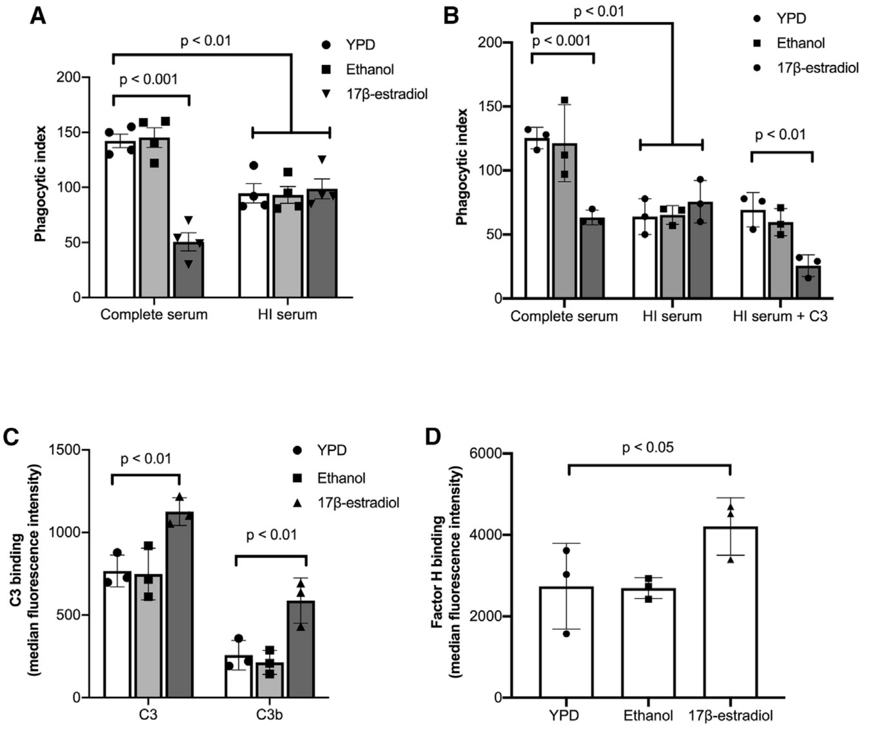

Figure 2. Estrogen promotes innate immune evasion of *C. albicans* through the inhibition of opsonophagocytosis

(A) J774A.1 macrophages were maintained in either complete serum or heat-inactivated (HI) serum and infected with *C. albicans* pre-exposed to YPD, 0.3% ethanol, or 10 μM 17β-estradiol at a multiplicity of infection (MOI) of 5.

(B) J774A.1 macrophages were maintained in either complete serum, heat-inactivated (HI) serum, or heat-inactivated serum supplemented with purified C3 and infected with *C. albicans* pre-exposed to YPD, 0.3% ethanol, or 10 μM 17β-estradiol at an MOI of 5.

(C) *C. albicans* was grown in YPD with or without 10 μM 17β-estradiol, incubated in human serum for 20 min, and fixed with 4% PFA, and C3 and C3b binding was quantified by fluorescence-activated cell sorting (FACS).

(D) *C. albicans* was grown in YPD with or without 10 μM 17β-estradiol, incubated in human serum for 20 min, and fixed with 4% PFA, and Factor H binding was quantified by FACS.

Data represent the mean ± SEM from at least three independent experiments. Individual data points represent each independent biological replicate. See also Figure S2.

induces altered expression of *C. albicans* cell surface proteins. To elucidate how quickly *C. albicans* adapts to estrogen, we grew the fungus in the presence of 10 μM 17β-estradiol for varying lengths of time before quantifying the macrophage phagocytosis rates. This time-course analysis confirmed that phagocytosis rates gradually reduced after 60 min of growth in the presence of estrogen (Figure 3A), suggesting that *C. albicans* undergoes some form of transcriptional or translational response to estrogen. To identify how estrogen affects the global transcriptional profile of *C. albicans*, we performed RNA sequencing (RNA-seq) on *C. albicans* cells that had been adapted to 10 μM 17β-estradiol for 4 h. Despite having a strong impact on the host-pathogen interaction, estrogen only had a mild effect on the transcriptome of *C. albicans*, with 59 genes being moderately upregulated and 75 genes being moderately downregulated in the presence of estrogen (Table S1, fold change >1.5, false discovery rate <0.05). Genes that were upregulated were largely involved in hormone binding, RNA processing, and flavin mononucleotide binding, while downregulated genes were largely involved in oxidoreductase activities (Table S2).

To identify whether the two differentially expressed genes involved in hormone binding (*EBP1* and *CDR1*) play a role in estrogen-induced innate immune evasion, we quantified the phagocytosis rates of the mutants in the presence and absence of estrogen. Deletion of *CDR1* did not affect estrogen-induced innate immune evasion (Figure 3B). However, deletion of *EBP1* resulted in consistently low phagocytosis even in the absence of hormone stimulation (Figure 3C), suggesting that Ebp1 is a negative regulator of innate immune evasion.

To identify whether estrogen mediates its effects through a conserved signaling pathway, we analyzed the promoter regions of the differentially expressed genes for the presence of conserved transcription factor binding motifs. From the top three putative motifs identified in the 5′ UTR of the differentially expressed genes (Table S3), 18 documented transcription factor binding motifs were identified (Table S4). Given that phagocytosis is governed by interactions between PAMPs located on the fungal cell surface and pattern recognition receptors on the phagocyte, we reasoned that enhanced Factor H binding and, therefore, innate immune evasion is likely to be mediated by proteins in the cell wall. From the 18 identified transcription factors, only Bcr1 has been linked to regulation of the fungal cell surface (Nobile and Mitchell, 2005). To identify whether Bcr1 plays a role in the estrogen response, we quantified phagocytosis rates of the *bcr1*Δ mutant in the presence and absence of estrogen. Deletion of *BCR1* resulted in the loss of innate immune evasion in the presence of estrogen (Figure 3D), confirming that Bcr1 is critical for this process.

To identify putative target genes of Bcr1, we analyzed gene expression data from Nobile and Mitchell (2005) in combination with data available on Pathoyeastract (Monteiro et al., 2019). In total, 45 genes were identified with documented Bcr1-binding sites in their promoters. To identify Bcr1 targets that function in the cell wall, we performed gene ontology (GO) term analysis on these 45 genes, whereby the top three GO terms are "cell surface," "plasma membrane," and "fungal-type cell wall" (Table S5). Of these cell surface associated genes, only Gpd2 has been linked to interaction with the complement system (Luo et al., 2012).

CellPress
OPEN ACCESS

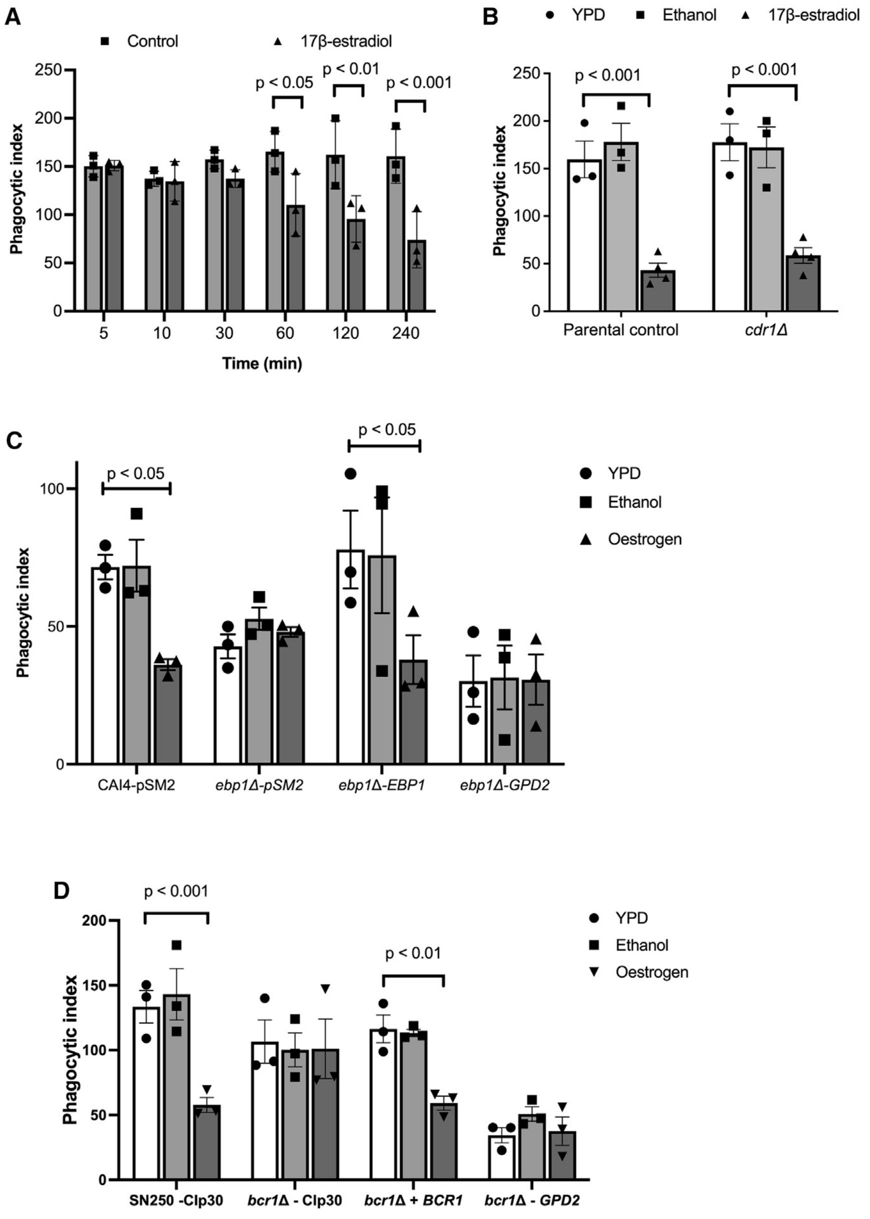

Figure 3. Estrogen induces transcriptional changes in fungal cell wall proteins through Ebp1 and Bcr1

(A) *C. albicans* was grown in YPD with or without 10 μM 17β-estradiol for increasing periods of time, cells were harvested, washed, and co-incubated with J774A.1 macrophages for 45 min, and phagocytosis rates were quantified. Data represent the mean ± SEM from three independent biological experiments.

(B) The *cdr1Δ* mutant and parental control strains were grown with or without 10 μM 17β-estradiol for 4 h and then exposed to J774A.1 macrophages for 45 min, and the phagocytosis rates were quantified.

(C) The *ebp1Δ* mutant, reconstituted strain, *GPD2* overexpression, and parental control strains were grown with or without 10 μM 17β-estradiol for 4 h and then exposed to J774A.1 macrophages for 45 min, and the phagocytosis rates were quantified.

(D) The *bcr1Δ* mutant, reconstituted strain, *GPD2* overexpression, and parental control strain were grown with or without 10 μM 17β-estradiol for 4 h and then exposed to J774A.1 macrophages for 45 min, and the phagocytosis rates were quantified.

All data represent the mean ± SEM from at least three independent biological experiments. Individual data points represent each independent biological replicate. See also Tables S1–S5.

Estrogen-induced innate immune evasion is mediated through Gpd2

GPD2 is a glycerol-3-phosphate dehydrogenase, which has been shown to localize to the fungal cell surface in response to serum (Marín et al., 2015). Gpd2 has been classed as a moonlighting protein (a protein that has multiple, but not linked biological functions) due to its ability to bind key regulatory components of the alternative complement system including Factor H, Factor H-like protein-1 (FHL-1), and plasminogen (Luo et al., 2012). Although not identified as differentially regulated by estrogen by RNA-seq, RT-PCR indicated that *GPD2* was mildly upregulated (1.9 ± SD 0.7-fold, p = 0.06) by estrogen. Thus, we hypothesized that estrogen-induced innate immune evasion of *C. albicans* is mediated via Gpd2-dependent acquisition of Factor H. To test this hypothesis, we deleted *GPD2* in *C. albicans* and determined the phagocytosis rates in the presence and absence of estrogen. Deletion of *GPD2* prevented estrogen-dependent inhibition of macrophage phagocytosis rates (Figure 4A). To determine whether the loss of innate immune evasion in the *gpd2*Δ mutant correlated with a reduction of Factor H recruitment to the fungal cell surface, we quantified Factor H binding in the *gpd2*Δ mutant in the presence and absence of estrogen. As predicted, the *gpd2*Δ mutant did not bind more Factor H than wild-type cells in the presence of estrogen (Figure 4B), confirming that immune evasion is dependent on Factor H recruitment. To confirm that enhanced expression of *GPD2* is sufficient to promote innate immune evasion, we overexpressed *GPD2* in *C. albicans*. Overexpression of *GPD2* (2.75-fold increased mRNA expression compared with wild-type cells) resulted in reduced phagocytosis rates irrespective of estrogen treatment (Figure 4C). The *GPD2* overexpression strain also bound more Factor H than the parental control strain (Figure 4D), thereby confirming that enhanced expression of *GPD2* is sufficient to promote *C. albicans* innate immune evasion. Given that deletion of *EBP1* results in constitutive innate immune evasion similar to the overexpression of *GPD2*, we quantified the expression of *GPD2* in the *ebp1*Δ mutant. Deletion of *EBP1*

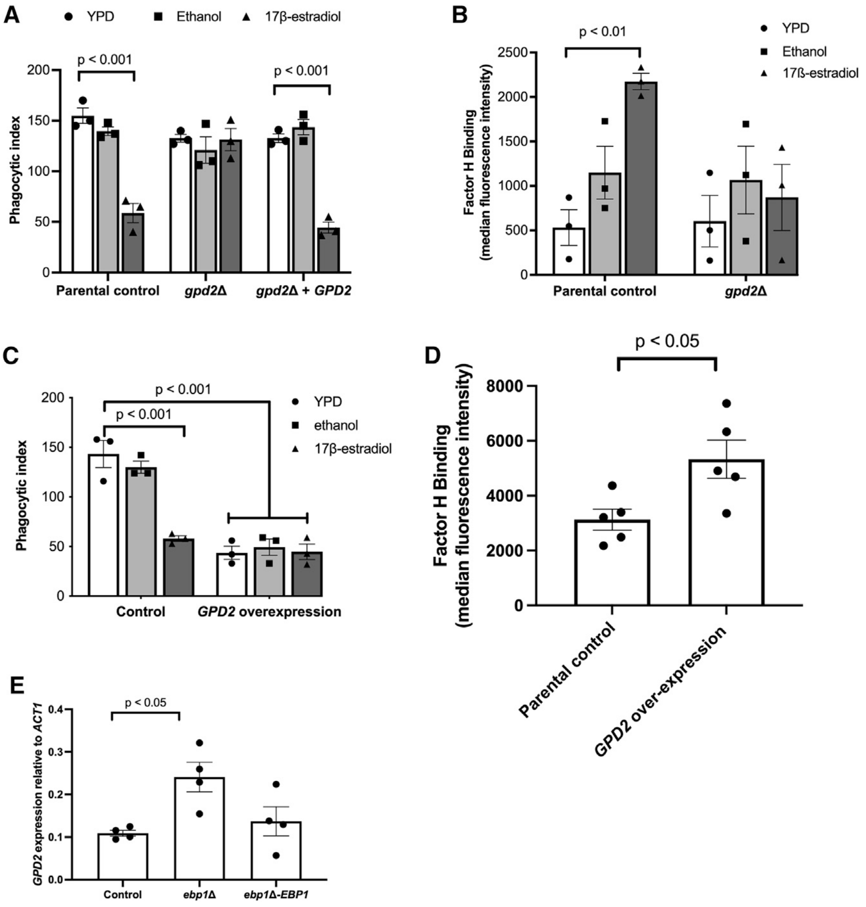

Figure 4. Inhibition of opsonophagocytosis is dependent on Gpd2

(A) The parental control strain, *gpd2*Δ mutant, and reconstituted control strains were grown in YPD with or without 10 μM 17β-estradiol for 4 h, and J77A.1 macrophage phagocytosis rates were quantified using ImageJ.

(B) *C. albicans* strains were grown in YPD with or without 10 μM 17β-estradiol, incubated in human serum for 20 min, and fixed with PFA, and Factor H binding was quantified by flow cytometry.

(C) The parental control (CAI4-pSM2) and *GPD2* overexpression (CAI4-pSM2-*GPD2*) strains were grown in YPD with or without 10 μM 17β-estradiol for 4 h, washed, and co-incubated with J774A.1 macrophages at an MOI of 5 for 45 min. Phagocytosis rates were quantified using ImageJ.

(D) The parental control (CAI4-pSM2) and *GPD2* overexpression (CAI4-pSM2-*GPD2*) strains were grown in YPD for 4 h, incubated in human serum for 20 min, and fixed with 4% PFA, and Factor H binding was quantified by flow cytometry.

(E) The *ebp1Δ* mutant, reconstituted control strain, and the parental control strain were grown in YPD to mid-log phase, total RNA extracted, and *GPD2* gene expression quantified by RT-PCR relative to *ACT1*.

All data represent the mean ± SEM from at least three independent biological experiments. Individual data points represent each independent biological replicate.

resulted in higher expression of *GPD2* compared with wild-type cells (Figure 4E), suggesting that Ebp1 is a negative regulator of *GPD2* expression and, therefore, innate immune evasion.

To identify whether Ebp1 and Bcr1 contribute to estrogen-induced innate immune evasion independent of regulating *GPD2* expression, we placed the expression of *GPD2* under the control of the *TEF2* promoter in the *ebp1*Δ and *bcr1*Δ mutants, resulting in the overexpression of *GPD2*. Overexpressing *GPD2* in the *ebp1*Δ mutant did not lead to significantly more innate immune evasion than in the *ebp1*Δ mutant (Figure 3C), suggesting that the defect in immune recognition of the *ebp1*Δ mutant stems from the elevated expression of *GPD2*. In addition, overexpression of *GPD2* in the *bcr1*Δ mutant restored innate immune evasion, confirming that the absence of immune evasion in the *bcr1*Δ mutant is a result of reduced *GPD2* expression (Figure 3D). Therefore, taken together these data indicate *GPD2* as a key player in estrogen-induced innate immune evasion.

Estrogen-induced immune evasion plays a key role in *C. albicans* pathogenicity

Having established that adaptation of *C. albicans* to estrogen inhibits phagocytosis, we explored whether this phenomenon could influence virulence *in vivo* using a zebrafish larval model for disseminated disease. Previously, it was shown that exposing zebrafish larvae (3 h post fertilization) to media containing 1 μM estrogen results in an *in vivo* estrogen concentration of 0.057 μM, equivalent to physiological levels during pregnancy in humans (Hao et al., 2013; Souder and Gorelick, 2017). Taking advantage of this observation, zebrafish larvae were infected with *C. albicans* SC5314 and maintained in E3 medium supplemented with 1 μM estrogen, and the survival rate was monitored for up to 5 days post fertilization. Compared with zebrafish incubated in E3 medium alone or E3 medium supplemented with ethanol, supplementation of E3 medium with 1 μM estrogen enhanced the virulence of *C. albicans*, resulting in a 63% reduction in zebrafish survival 60 h after infection (Figure 5A).

To investigate whether Gpd2 is required for virulence in this model, we infected zebrafish with the parental control strain, the *gpd2*Δ mutant, or reconstituted control strains of *C. albicans*. Deletion of *GPD2* led to attenuation of *C. albicans* virulence, which was restored to parental control levels via complementation with a single copy of *GPD2* (Figure 5B). To assess whether increased expression of Gpd2 is sufficient to drive fungal virulence, we infected zebrafish larvae with a *C. albicans* strain that overexpresses *GPD2* in the absence of estrogen stimulation. Overexpression of *GPD2* enhanced *C. albicans* virulence compared with the respective control strain (Figure 5C). Therefore, Gpd2 plays a key role in promoting *C. albicans* virulence *in vivo*.

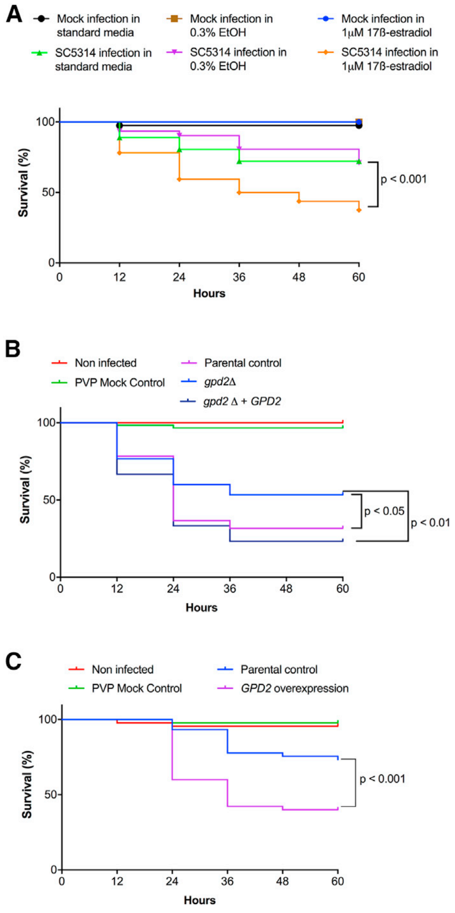

Figure 5. Estrogen promotes the virulence of *C. albicans* in a zebrafish larval infection model

(A) *C. albicans* (SC5314) was microinjected into the hindbrain ventricle of zebrafish larvae in the Prim25 stage. Infected larvae were maintained in E3 medium, or E3 medium supplemented with 0.3% ethanol, or 1 μM 17β-estradiol, and larval survival was monitored every 24 h until day 5 post fertilization.

(B) The parental control (SN250-CIP30), *gpd2Δ* mutant (*gpd2Δ*-CIP30), and reconstituted control (*gpd2Δ*-CIP30-*GPD2*) strains were microinjected into the hindbrain ventricle of zebrafish larvae in the Prim25 stage. Larvae were maintained in E3 medium and larval survival was monitored every 24 h until day 5 post fertilization.

(C) The parental control (CAI4-pSM2) or *GDP2* overexpression (CAI4-pSM2-*GPD2*) strains were microinjected into the hindbrain ventricle of zebrafish larvae in the Prim25 stage. Larvae were maintained in E3 medium and larval survival was monitored every 24 h until day 5 post fertilization.

The survival curves represent data pooled from three independent experiments. Statistically significant differences were determined by the log rank (Mantel-Cox) test.

DISCUSSION

VVC is a mucosal infection affecting 75% of the female population of reproductive age (Sobel, 1988). Estrogen is known to govern susceptibility to VVC infections, with women with low circulatory estrogen levels (i.e., postmenopausal women) having a low risk of developing VVC, and women with high estrogen levels (i.e., during pregnancy, or women taking high-estrogen-containing oral contraceptives) having a high risk of VVC. Elevated estrogen levels increase glycogen at the vaginal mucosa, reduce leukocyte infiltration, and reduce antifungal activity of epithelial cells, promoting infection (Fidel et al., 2000; Dennerstein and Ellis, 2001; Salinas-Muñoz et al., 2018). However, here we show that, in addition to these effects on the host, estrogen promotes adaptation responses in *C. albicans* which induce evasion of the innate immune system through Gpd2-dependent inhibition of complement-mediated opsonophagocytosis. Unlike the induction of fungal morphogenesis, which appears to be limited to 17β-estradiol (Cheng et al., 2006), all tested forms of estrogen promoted innate immune evasion, suggesting that *C. albicans* has evolved at least two signaling pathways that are responsive to estrogen.

Complement evasion is a successful mechanism employed by viruses, bacteria, parasites, and fungi to escape the innate immune system (Lambris et al., 2008). Evasion of the complement system can be mediated through the secretion of degradative proteins or by recruitment of host regulatory proteins (Merle et al., 2015). One of the most common methods used by pathogens to evade complement is through enhanced recruitment of Factor H, a key regulatory protein in the complement system, to the microbial surface. Bound Factor H prevents further activation of the alternative complement system through both the destabilization of the C3 convertase and enhancement of Factor I-mediated degradation of C3b to iC3b, reducing opsonization and inhibiting the formation of the membrane attack complex (Merle et al., 2015). *C. albicans* has been shown, by others, to recruit Factor H to its surface through the expression of moonlighting proteins. Moonlighting proteins are proteins that perform multiple, unlinked biological functions. So far, four moonlighting proteins (Phr1, Gpd2, Hgt1, and Gpm1) have been identified in *C. albicans* (Poltermann et al., 2007; Luo et al., 2010, 2012; Kenno et al., 2018), but only *GPD2* was regulated by estrogen, suggesting that the other moonlighting proteins may mediate innate immune evasion in response to other environmental stimuli. Although Gpd2 is predicted to be a cytoplasmic protein involved in glycerol production, Gpd2 has also been identified in cell wall proteomic studies (Marín et al., 2015), suggesting that Gpd2 also functions in the cell wall. Interestingly, Gpd2 was only identified in the cell wall proteome of *C. albicans* grown in complete serum and not heat-inactivated serum where complement proteins have been inactivated (Marín et al., 2015). Therefore, deposition of complement on the fungal cell surface may drive localization of Gpd2 to the cell surface.

Purified Gpd2 binds all three complement regulatory proteins (Luo et al., 2012), although the biological significance of these interactions in infections is not known. Factor H and FHL1 bound to Gpd2 remain active, cleaving C3b and thereby inhibiting the alternative complement system (Luo et al., 2012). In addition, Gpd2 can also bind plasminogen, which is then processed into plasmin, contributing to inactivation of the alternative complement system (Luo et al., 2012). Flow cytometry confirmed that estrogen-adapted cells bound more Factor H, indicative of estrogen promoting complement evasion. However, estrogen-adapted *C. albicans* also bound more C3 and C3b, which is surprising, as binding of Factor H should prevent C3 deposition. Factor H is a glycoprotein that is made up of 20 complement

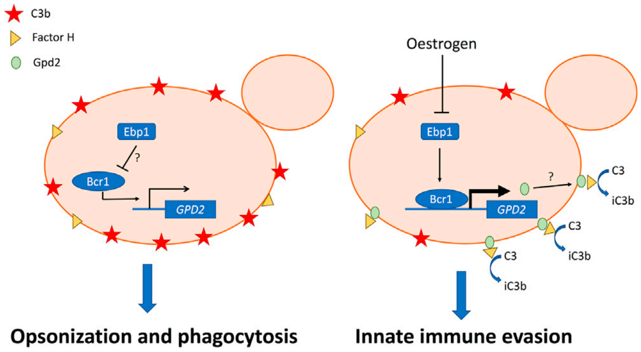

Figure 6. Proposed mechanism of estrogen-induced innate immune evasion

Under standard laboratory conditions the C3 is deposited on the surface of *C. albicans*, resulting in effective phagocytosis. However, in the presence of estrogen the NADPH activity of Ebp1 is reduced, resulting in derepression (through an as yet to be identified signaling pathway) and surface localization of Gpd2. Gpd2 recruits Factor H to the fungal cell surface, preventing the formation of the C3 convertase and disposition of C3 on the fungal cell surface. Reduced opsonization results in innate immune evasion and insufficient clearance of *C. albicans*, promoting fungal pathogenicity.

control proteins (CCPs). Microbes bind Factor H at various positions, with *Neisseria* species binding Factor H at CCP6–8 (Ngampasutadol et al., 2008). CCP7 has been shown to be important for the interaction of Factor H with recombinant Gpd2 (Luo et al., 2012). However, multiple microbes bind Factor H at CCP20, and this has been termed the common microbial binding site (Meri et al., 2013). Binding of Factor H to some of these microbial cell surface proteins at CCP20 results in increased affinity of Factor H for C3b (Meri et al., 2013). The enhanced affinity of Factor H for C3b might explain why we observe elevated binding of both C3b and Factor H, and would suggest that *in vivo* CCP20 may play an important role in the interaction between Factor H and Gpd2. The formation of this stable tripartite complex (microbial protein, Factor H, and C3b) results in enhanced activity of Factor H and therefore rapid inactivation of the alternative complement cascade (Meri et al., 2013), which would explain why we observe reduced levels of phagocytosis (Figure 6).

Innate immune evasion was only observed after *C. albicans* had been grown in the presence of estrogen for 60 min, suggesting that the response required either transcriptional or post-transcriptional regulation. However, in line with previous studies (Cheng et al., 2006), global transcriptional analysis confirmed that only a small proportion of *C. albicans* genes were differentially regulated by estrogen. In comparison with the work of Cheng et al. (2006), in which most of the differentially regulated genes are involved in fungal morphogenesis, our analysis mainly identified genes involved in metabolism and oxidation-reduction processes. This lack of overlap is likely reflective of the growth conditions used, as Cheng et al. used RPMI medium whereby estrogen promotes hyphal formation (Cheng et al., 2006; Salinas-Muñoz et al., 2018), while under our experimental conditions (YPD medium) estrogen did not promote hyphal formation. Although innate immune evasion was dependent on the presence of Gpd2, *GPD2* mRNA levels were only moderately increased in response to estrogen (1.9 ± 0.7-fold), suggesting that other factors (i.e., post-translational modifications, protein localization), in combination with gene regulation, may be involved. Given that Gpd2 has only been identified as a cell wall protein under specific conditions (Marín et al., 2015), it might be that in response to estrogen a higher level of Gpd2 is recruited to the cell wall, enabling the fungus to bind more Factor H. In *Saccharomyces cerevisiae*, the activity of both Gpd1 and Gpd2 is regulated via post-translational modification (Lee et al., 2012). Snf1-dependent phosphorylation of *Sc*Gpd1 (ortholog of Ca*GPD2*) results in decreased enzyme activity (Lee et al., 2012). Although *Ca*Gpd2 has several predicted phosphorylation sites, these are located in different regions of the protein compared with *Sc*Gpd1, and therefore it is possible that phosphorylation/dephosphorylation at any of these sites in *Ca*Gpd2 could affect the localization or potential to bind Factor H.

Ebp1, despite showing no homology to mammalian hormone binding proteins, has been described as an estrogen-binding protein. Ebp1 is homologous to old yellow enzyme 2 (*OYE2*) of *S. cerevisiae*, an NADPH oxidoreductase. Biochemical characterization of recombinant Ebp1 confirms that Ebp1 is an NADPH oxidoreductase and that estrogen inhibits this enzymatic activity (Madani et al., 1994; Buckman and Miller, 1998). The importance of Ebp1 enzymatic activity in *C. albicans* pathogenicity is not known, but here we demonstrate that Ebp1 may serve as a negative regulator of innate immune evasion through the regulation of *GPD2*. Interaction of estrogen with Ebp1, through an as yet unidentified pathway likely involving Bcr1, results in elevated expression of *GPD2* and, potentially in association with other post-translational modifications, promotes innate immune evasion and pathogenicity (Figure 6).

In addition to estrogen, a variety of other compounds that contain a phenolic group have been shown to affect the activity of Ebp1 (Buckman and Miller, 1998), suggesting that these compounds may also affect the immune recognition of *C. albicans* through differential regulation of *GPD2*. However, progesterone, which contains an unsaturated α,β-keto structure similar to estrogen, did not affect innate immune recognition of *C. albicans*. To date, Ebp1 has not been shown to be inhibited by progesterone. Therefore, it is possible that subtle differences in the chemical composition of the two hormones determine their interaction with the Ebp1 active site. However, biochemical studies, in combination with structure-activity relationship studies, are needed to identify specifically which molecules are capable of inhibiting Ebp1.

In recent years zebrafish larvae have become an excellent model for studying pathogenicity mechanisms. Zebrafish have a complement system that is structurally and functionally similar to the mammalian complement system (Zhang and Cui, 2014), and an ortholog of Factor H has been identified and cloned (Sun et al., 2010). The presence of estrogen in the zebrafish larval model of disseminated *C. albicans* infection confirmed that estrogen promotes the virulence of *C. albicans*, suggesting that estrogen-dependent inactivation of the alternative complement system promotes the virulence of *C. albicans in vivo*. These data would suggest that women with elevated estrogen levels would be more prone to systemic infections as well as vaginal infections, which has not been documented. Systemic and mucosal infections elicit different immune responses, and these immune

CellPress
OPEN ACCESS

responses are niche specific. VVC is a very complex infection, with a variety of environmental factors contributing to the development of infection. The vaginal epithelial cells together with infiltrating neutrophils are important innate immune cells for maintaining *C. albicans* colonization levels and preventing symptomatic infection. During VVC, neutrophil function is already diminished through the production of heparin sulfate, and epithelial cells display less antifungal activity (Yano et al., 2018). Therefore, we propose that estrogen-induced inhibition of opsonophagocytosis is just one of several mechanisms that predispose women to VVC. Given the strong association of elevated estrogen levels with the development of VVC, it is important to understand the role of Gpd2 in promoting innate immune evasion, which may lead to the development of alternative treatment options for VVC and RVVC.

Limitations of the study

One limitation of this study is the focus on yeast cells grown in rich media. In the host, *C. albicans* grows as yeast, pseudohyphae, and true hyphae, and all these morphologies contribute to fungal virulence. Although Gpd2 is expressed in hyphae, we have not directly confirmed that Gpd2 functions in the same manner in hyphal cells. Therefore, investigating the role of morphogenesis in this immune evasion strategy is a key next step. In addition, host-specific environmental cues alter the composition of the cell wall, affecting innate immune recognition. Although estrogen itself does not affect the cell wall composition, we have not investigated whether *C. albicans* adapts to estrogen similarly in different growth media. However, estrogen did promote virulence in a zebrafish larval model of disseminated infection, but again we acknowledge that this infection model does not recapitulate VVC infections.

STAR★METHODS

Detailed methods are provided in the online version of this paper and include the following:

- KEY RESOURCES TABLE
- RESOURCE AVAILABILITY
  - Lead contact
  - Materials availability
  - Data and code availability
- EXPERIMENTAL MODEL AND SUBJECT DETAILS
  - Fungi
  - Cell lines
  - Zebrafish
  - Human macrophages and monocytes
- METHOD DETAILS
  - Phagocytosis experiments
  - Human macrophages and neutrophils
  - Genetic manipulation of C. albicans
  - *Immunofluorescent staining of* C. albicans *cell wall components*
  - RNA sequencing
  - RT-PCR
  - Complement binding
  - Zebrafish infection
- QUANTIFICATION OF STATISTICAL ANALYSIS

SUPPLEMENTAL INFORMATION

Supplemental information can be found online at https://doi.org/10.1016/j.celrep.2021.110183.

ACKNOWLEDGMENTS

We would like to thank the fungal community for the donation of fungal strains, Prof. G. Brown (University of Exeter) for FC-Dectin-1, Prof. G. Ramage for clinical isolates, the Environmental Omics facility at the University of Birmingham for help with the RNA sequencing, as well as Elizabeth Ballou (University of Birmingham) for critical reading of the manuscript, and Prof. A. Mitchell for providing access to raw data from Nobile and Mitchell (2005). P.K. is funded by the Wellcome Trust strategic award for medical mycology (097377/Z/11/Z), F.C. and R.A.H. are funded by the MRC (MR/L00903X/1) and BBSRC (BB/R00966X/1), A.C.H. is funded by the BBSRC (BB/R00966X/2), and D.K. is funded by the Graduate Training Program at the University of Kent.

AUTHOR CONTRIBUTIONS

P.K., F.C., B.K., H.G., A.C.H., and D.K. conducted the experiments; F.C. and H.-J.T. conducted the bioinformatics analysis; R.A.H. conceived the idea and designed the experiments; R.A.H. wrote the paper.

DECLARATION OF INTERESTS

The authors declare no competing interests.

INCLUSION AND DIVERSITY

We worked to ensure gender balance in the recruitment of human subjects. We worked to ensure ethnic or other types of diversity in the recruitment of human subjects. We worked to ensure that the study questionnaires were prepared in an inclusive way.

One or more of the authors of this paper self-identifies as an underrepresented ethnic minority in science. One or more of the authors of this paper received support from a program designed to increase minority representation in science. The author list of this paper includes contributors from the location where the research was conducted who participated in the data collection, design, analysis, and/or interpretation of the work.

Received: September 11, 2020
Revised: October 1, 2021
Accepted: December 7, 2021
Published: January 4, 2022

REFERENCES

Amirshahi, A., Wan, C., Beagley, K., Latter, J., Symonds, I., and Timms, P. (2011). Modulation of the *Chlamydia trachomatis* in vitro transcriptome response by the sex hormones estradiol and progesterone. BMC Microbiol. *11*, 150.

Ballou, E.R., Avelar, G.M., Childers, D.S., Mackie, J., Bain, J.M., Wagener, J., Kastora, S.L., Panea, M.D., Hardison, S.E., Walker, L.A., et al. (2016). Lactate signalling regulates fungal β-glucan masking and immune evasion. Nat. Microbiol. *2*, 16238.

Barkani, A.E., Kurzai, O., Fonzi, W.A., Porta, R.A., Frosch, M., and Muhlschlegel, F.A. (2000). Dominant active alleles of *RIM101* (*PRR2*) bypass the pH restriction on filamentation of *Candida albicans*. Mol. Cell. Biol. *20*, 4635–4647.

Buckman, J., and Miller, S.M. (1998). Binding and reactivity of *Candida albicans* estrogen binding protein with steroid and other substrates. Biochemistry *37*, 14326–14336.

Cheng, G., Yeater, K.M., and Hoyer, L.L. (2006). Cellular and molecular biology of *Candida albicans* estrogen response. Eukaryot. Cell *5*, 180–191.

Chotirmall, S.H., Smith, S.G., Gunaratnam, C., Cosgrove, S., Dimitrov, B.D., O'Neill, S.J., Harvey, B.J., Greene, C.M., and McElvaney, N.G. (2012). Effect

of estrogen on *Pseudomonas* mucoidy and exacerbations in cystic fibrosis. N. Engl. J. Med. *366*, 1978–1986.

Cottier, F., Sherrington, S., Cockerill, S., del Olmo Toledo, V., Kissane, S., Tournu, H., Orsini, L., Palmer, G.E., Pérez, J.C., and Hall, R.A. (2019). Remasking of *Candida albicans* β-glucan in response to environmental pH is regulated by quorum sensing. mBio *10*. https://doi.org/10.1128/mBio.02347-19.

Dasari, P., Shopova, I.A., Stroe, M., Wartenberg, D., Martin-Dahse, H., Beyersdorf, N., Hortschansky, P., Dietrich, S., Cseresnyés, Z., Figge, M.T., et al. (2018). Aspf2 from *Aspergillus fumigatus* recruits human immune regulators for immune evasion and cell damage. Front. Immunol. *9*, 1635.

Dennerstein, G.J., and Ellis, D.H. (2001). Oestrogen, glycogen and vaginal candidiasis. Aust. N.Z. J. Obstet. Gynecol. *41*, 326–328.

Dennison, P.M.J., Ramsdale, M., Manson, C.L., and Brown, A.J.P. (2005). Gene disruption in *Candida albicans* using a synthetic, codon-optimised Cre-loxP system. Fungal Genet. Biol. *42*, 737–748.

Fidel, P.L., Jr., Cutright, J., and Steele, C. (2000). Effects of reproductive hormones on experimental vaginal candidiasis. Infect. Immun. *68*, 651–657.

Fonzi, W.A., and Irwin, M.Y. (1993). Isogenic strain construction and gene mapping in *Candida albicans*. Genetics *134*, 717–728.

García-Gómez, E., González-Pedrajo, B., and Camacho-Arroyo, I. (2013). Role of sex steroid hormones in bacterial-host interactions. Biomed. Res. Int. *2013*, 928290.

Ghazeeri, G., Abdullah, L., and Abbas, O. (2011). Immunological differences in women compared with men: overview and contributing factors. Am. J. Reprod. Immunol. *66*, 163–169.

Gillum, A.M., Tsay, E.Y.H., and Kirsch, D.R. (1984). Isolation of the *Candida albicans* gene for orotidine-5′-phosphate decarboxylase by complementation of *S. cerevisiae ura3* and *E. coli* pyrF mutations. Mol. Gen. Genet. *198*, 179–182.

Gonçalves, B., Ferreira, C., Alves, C.T., Henriques, M., Azeredo, J., and Silva, S. (2016). Vulvovaginal candidiasis: epidemiology, microbiology and risk factors. Crit. Rev. Microbiol. *42*, 905–927.

György, C. (2017). Is there a hormonal regulation of phagocytosis at unicellular and multicellular levels? A critical review. Acta Microbiol. Immunol. Hung. *64*, 357–372.

Hall, R.A. (2015). Dressed to impress: impact of environmental adaptation on the *Candida albicans* cell wall. Mol. Microbiol. *97*, 7–17.

Hall, R.A., and Gow, N.A.R. (2013). Mannosylation in *Candida albicans*: role in cell wall function and immune recognition. Mol. Microbiol. *90*, 1147–1161.

Hall, R.A., De Sordi, L., MacCallum, D.M., Topal, H., Eaton, R., Bloor, J.W., Robinson, G.K., Levin, L.R., Buck, J., Wang, Y., et al. (2010). CO_2 acts as a signalling molecule in populations of the fungal pathogen *Candida albicans*. PLoS Pathog. *6*, e1001193.

Hao, R., Bondesson, M., Singh, A.V., Riu, A., McCollum, C.W., Knudsen, T.B., Gorelick, D.A., and Gustafsson, J.-Å. (2013). Identification of estrogen target genes during zebrafish embryonic development through transcriptomic analysis. PLoS One *8*, e79020.

Hopke, A., Nicke, N., Hidu, E.E., Degani, G., Popolo, L., and Wheeler, R.T. (2016). Neutrophil attack triggers extracellular trap-dependent *Candida* cell wall remodeling and altered immune recognition. PLoS Pathog. *12*, e1005644.

Inglis, D.O., Arnaud, M.B., Binkley, J., Shah, P., Skrzypek, M.S., Wymore, F., Binkley, G., Miyasato, S.R., Simison, M., and Sherlock, G. (2012). The *Candida* genome database incorporates multiple *Candida* species: multispecies search and analysis tools with curated gene and protein information for *Candida albicans* and *Candida glabrata*. Nucleic Acids Res. *40*, D667–D674.

Kenno, S., Speth, C., Rambach, G., Binder, U., Chatterjee, S., Caramalho, R., Haas, H., Lass Flörl, C., Shaughnessy, J., and Ram, S. (2018). *Candida albicans* factor H binding molecule Hgt1p—a low glucose-induced transmembrane protein is trafficked to the cell wall and impairs phagocytosis and killing by human neutrophils. Front. Microbiol. *9*, 3319.

Kinsman, O.S., and Collard, A.E. (1986). Hormonal factors in vaginal candidiasis in rats. Infect. Immun. *53*, 498–504.

Klein, S.L. (2000). The effects of hormones on sex differences in infection: from genes to behavior. Neurosci. Biobehavioral Rev. *24*, 627–638.

Kozel, T.R., Weinhold, L.C., and Lupan, D.M. (1996). Distinct characteristics of initiation of the classical and alternative complement pathways by *Candida albicans*. Infect. Immun. *64*, 3360–3368.

Lambris, J.D., Ricklin, D., and Geisbrecht, B.V. (2008). Complement evasion by human pathogens. Nat. Rev. Microbiol. *6*, 132–142.

Lee, Y.J., Jeschke, G.R., Roelants, F.M., Thorner, J., and Turk, B.E. (2012). Reciprocal phosphorylation of yeast glycerol-3-phosphate dehydrogenases in adaptation to distinct types of stress. Mol. Cell. Biol. *32*, 4705–4717.

Lopes, J.P., Stylianou, M., Backman, E., Holmberg, S., Jass, J., Claesson, R., and Urban, C.F. (2018). Evasion of immune surveillance in low oxygen environments enhances *Candida albicans* virulence. mBio *9*. https://doi.org/10.1128/mBio.02120-18.

van Lunzen, J., and Altfeld, M. (2014). Sex differences in infectious diseases-common but neglected. J. Infect. Dis. *209*, S79–S80.

Luo, S., Hartmann, A., Dahse, H.-M., Skerka, C., and Zipfel, P.F. (2010). Secreted pH-regulated antigen 1 of *Candida albicans* blocks activation and conversion of complement C3. J. Immunol. *185*, 2164–2173.

Luo, S., Hoffmann, R., Skerka, C., and Zipfel, P.F. (2012). Glycerol-3-phosphate dehydrogenase 2 is a novel factor H-, factor H-like protein 1-, and plasminogen-binding surface protein of *Candida albicans*. J. Infect. Dis. *207*, 594–603.

Madani, N.D., Malloy, P.J., Rodriguez-Pombo, P., Krishnan, A.V., and Feldman, D. (1994). *Candida albicans* estrogen-binding protein gene encodes an oxidoreductase that is inhibited by estradiol. Proc. Natl. Acad. Sci. U S A *91*, 922–926.

Mallick, E.M., Bergeron, A.C., Jones, S.K., Newman, Z.R., Brothers, K.M., Creton, R., Wheeler, R.T., and Bennett, R.J. (2016). Phenotypic plasticity regulates *Candida albicans* interactions and virulence in the vertebrate host. Front. Microbiol. *7*. https://doi.org/10.3389/fmicb.2016.00780.

Marín, E., Parra-Giraldo, C.M., Hernández-Haro, C., Hernáez, M.L., Nombela, C., Monteoliva, L., and Gil, C. (2015). *Candida albicans* shaving to profile human serum proteins on hyphal surface. Front. Microbiol. *6*, 1343.

van der Maten, E., van den Broek, B., de Jonge, M.I., Rensen, K.J.W., Eleveld, M.J., Zomer, A.L., Cremers, A.J.H., Ferwerda, G., de Groot, R., Langereis, J.D., et al. (2018). *Streptococcus pneumoniae* PspC subgroup prevalence in invasive disease and differences in contribution to complement evasion. Infect. Immun. *86*, e00010–00018.

McClelland, E.E., and Smith, J.M. (2011). Gender specific differences in the immune response to infection. Arch. Immunol. Ther. Exp. (Warsz) *59*, 203–213.

Meri, T., Hartmann, A., Lenk, D., Eck, R., Würzner, R., Hellwage, J., Meri, S., and Zipfel, P.F. (2002). The yeast *Candida albicans* binds complement regulators factor H and FHL-1. Infect. Immun. *70*, 5185–5192.

Meri, T., Amdahl, H., Lehtinen, M.J., Hyvärinen, S., McDowell, J.V., Bhattacharjee, A., Meri, S., Marconi, R., Goldman, A., and Jokiranta, T.S. (2013). Microbes bind complement inhibitor factor H via a common site. PLoS Pathog. *9*, e1003308.

Merle, N.S., Noe, R., Halbwachs-Mecarelli, L., Fremeaux-Bacchi, V., and Roumenina, L.T. (2015). Complement system part II: role in immunity. Front. Immunol. *6*, 257.

Monteiro, P.T., Oliveira, J., Pais, P., Antunes, M., Palma, M., Cavalheiro, M., Galocha, M., Godinho, C.P., Martins, L.C., Bourbon, N., et al. (2019). YEASTRACT+: a portal for cross-species comparative genomics of transcription regulation in yeasts. Nucleic Acids Res. *48*, D642–D649.

Netea, M.G., Brown, G.D., Kullberg, B.J., and Gow, N.A.R. (2008). An integrated model of the recognition of *Candida albicans* by the innate immune system. Nat. Rev. Microbiol. *6*, 67–78.

Ngampasutadol, J., Ram, S., Gulati, S., Agarwal, S., Li, C., Visintin, A., Monks, B., Madico, G., and Rice, P.A. (2008). Human factor H interacts selectively with *Neisseria gonorrhoeae* and results in species-specific complement evasion. J. Immunol. *180*, 3426.

CellPress
OPEN ACCESS

Nobile, C.J., and Mitchell, A.P. (2005). Regulation of cell-surface genes and biofilm formation by the *C. albicans* transcription factor Bcr1p. Curr. Biol. *15*, 1150–1155.

Noble, S.M., French, S., Kohn, L.A., Chen, V., and Johnson, A.D. (2010). Systematic screens of a *Candida albicans* homozygous deletion library decouple morphogenetic switching and pathogenicity. Nat. Genet. *42*, 590–598.

Pericolini, E., Perito, S., Castagnoli, A., Gabrielli, E., Mencacci, A., Blasi, E., Vecchiarelli, A., and Wheeler, R.T. (2018). Epitope unmasking in vulvovaginal candidiasis is associated with hyphal growth and neutrophilic infiltration. PLoS One *13*, e0201436.

Poltermann, S., Kunert, A., von der Heide, M., Eck, R., Hartmann, A., and Zipfel, P.F. (2007). Gpm1p is a factor H-, FHL-1-, and plasminogen-binding surface protein of *Candida albicans*. J. Biol. Chem. *282*, 37537–37544.

Pradhan, A., Avelar, G.M., Bain, J.M., Childers, D.S., Larcombe, D.E., Netea, M.G., Shekhova, E., Munro, C.A., Brown, G.D., Erwig, L.P., et al. (2018). Hypoxia promotes immune evasion by triggering β-glucan masking on the *Candida albicans* cell surface via mitochondrial and cAMP-protein kinase A signaling. mBio *9*, e01318–01318.

Pradhan, A., Avelar, G.M., Bain, J.M., Childers, D., Pelletier, C., Larcombe, D.E., Shekhova, E., Netea, M.G., Brown, G.D., Erwig, L., et al. (2019). Non-canonical signalling mediates changes in fungal cell wall PAMPs that drive immune evasion. Nat. Commun. *10*, 5315.

Salinas-Muñoz, L., Campos-Fernández, R., Mercader, E., Olivera-Valle, I., Fernández-Pacheco, C., Matilla, L., García-Bordas, J., Brazil, J.C., Parkos, C.A., Asensio, F., et al. (2018). Estrogen receptor-alpha (*ESR1*) governs the lower female reproductive tract vulnerability to *Candida albicans*. Front. Immunol. *9*, 1033.

Sanglard, D., Ischer, F., Monod, M., and Bille, J. (1997). Cloning of *Candida albicans* genes conferring resistance to azole antifungal agents: characterization of *CDR2*, a new multidrug ABC transporter gene. Microbiology *143* (*Pt 2*), 405–416.

Sherrington, S.L., Sorsby, E., Mahtey, N., Kumwenda, P., Lenardon, M.D., Brown, I., Ballou, E.R., MacCallum, D.M., and Hall, R.A. (2017). Adaptation of *Candida albicans* to environmental pH induces cell wall remodelling and enhances innate immune recognition. PLoS Pathog. *13*, e1006403.

Sobel, J.D. (1988). Pathogenesis and epidemiology of vulvovaginal candidiasis. Ann. New York Acad. Sci. *544*, 547–557.

Sobel, J.D. (1997). Vaginitis. N. Engl. J. Med. *337*, 1896–1903.

Souder, J.P., and Gorelick, D.A. (2017). Quantification of estradiol uptake in zebrafish embryos and larvae. Toxicol. Sci. *158*, 465–474.

Sun, G., Li, H., Wang, Y., Zhang, B., and Zhang, S. (2010). Zebrafish complement factor H and its related genes: Identification, evolution, and expression. Funct. Integr. Genomics *10*, 577–587.

Taneja, V. (2018). Sex hormones determine immune response. Front. Immunol. *9*. https://doi.org/10.3389/fimmu.2018.01931.

Tripathi, A., Liverani, E., Tsygankov, A., and Puri, S. (2020). Iron alters the cell wall composition and intracellular lactate to affect *Candida albicans* susceptibility to antifungals and host immune response. J. Biol. Chem. *295*, 10032–10044.

Walther, A., and Wendland, J. (2003). An improved transformation protocol for the human fungal pathogen *Candida albicans*. Curr. Genet. *42*, 339–343.

Wheeler, R.T., and Fink, G.R. (2006). A drug-sensitive genetic network masks fungi from the immune system. Plos Pathog. *2*, e35.

Wheeler, R.T., Kombe, D., Agarwala, S.D., and Fink, G.R. (2008). Dynamic, morphotype-specific *Candida albicans* β-glucan exposure during infection and drug treatment. PLoS Pathog. *4*, e1000227.

White, S., and Larsen, B. (1997). *Candida albicans* morphogenesis is influenced by estrogen. Cell. Mol. Life Sci. *53*, 744–749.

Williams, R.B., and Lorenz, M.C. (2020). Multiple alternative carbon pathways combine to promote *Candida albicans* stress resistance, immune interactions, and virulence. mBio *11*. https://doi.org/10.1128/mBio.03070-19.

Wilson, R.B., Davis, D., Enloe, B.M., and Mitchell, A.P. (2000). A recyclable *Candida albicans URA3* cassette for PCR product-directed gene disruptions. Yeast *16*, 65–70.

Yano, J., Peters, B.M., Noverr, M.C., and Fidel, P.L., Jr. (2018). Novel mechanism behind the immunopathogenesis of vulvovaginal candidiasis: neutrophil anergy. Infect. Immun. *86*. https://doi.org/10.1128/IAI.00684-17.

Zhang, S., and Cui, P. (2014). Complement system in zebrafish. Dev. Comp. Immunol. *46*, 3–10.

STAR★METHODS

KEY RESOURCES TABLE

REAGENT or RESOURCE	SOURCE	IDENTIFIER
**Antibodies**		
Anti-Factor H Goat pAb	Sigma	341276-1ML; RRID:AB_211828
Rabbit anti Goat IgG (H+L) Alexa Fluor 488	Invitrogen	A11078; RRID:AB_2534122
Goat anti Chicken IgY (H+L) Alexa Fluor 594	Invitrogen	A11042; RRID:AB_2534099
Anti-complement C3b/iC3b antibody clone 6C9	Millipore	MABF982
Anti-complement C3 Antibody	Millipore	AB3421; RRID:AB_240685
**Bacterial and virus strains**		
SC5314: wildtype	(Gillum et al., 1984)	N/A
SVS006B: clinical isolate	Prof Ramage, Glasgow University	N/A
SVS062A: clinical isolate	Prof Ramage, Glasgow University	N/A
SN152: *arg4Δ/arg4Δ leu2Δ/leu2Δ his1Δ/his1Δ URA3/ura3Δ::λimm434 IRO1/iro1Δ:: λimm434*	(Noble et al., 2010)	N/A
SN250: *his1Δ/his1Δ*, *leu2Δ::C. dubliniensis HIS1 /leu2Δ::C. maltosa LEU2-arg4Δ/arg4Δ*, *URA3/ura3Δ::imm434-IRO1/iro1Δ::imm434*	(Noble et al., 2010)	N/A
SN250-CIP30: As SN250 but *RPS1/rps1*::CIP30	This study	N/A
*rob1*Δ: As SN152 but *rob1Δ::C. dubliniensisHIS1/rob1Δ::C. maltose LEU2*	(Noble et al., 2010)	N/A
*bcr1*Δ: *arg4Δ/arg4Δ leu2Δ/leu2Δ his1Δ/his1Δ URA3/ura3Δ::λimm434 IRO1/iro1Δ:: λimm434 bcr1::LEU/bcr1::HIS1*	(Noble et al., 2010)	N/A
*gpd2*Δ: As SN152 but *gpd2Δ::C. dubliniensisHIS1/gpd2Δ::C. maltose LEU2*	(Noble et al., 2010)	N/A
*gpd2*Δ–CIP30: As SN152 but *gpd2Δ::C. dubliniensisHIS1/gpd2Δ::C. maltose LEU2*, *RPS1/rps1::CIP30*	This study	N/A
*gpd2*Δ-CIP30-*GPD2*: As SN152 but *gpd2Δ::C. dubliniensisHIS1/gpd2Δ::C. maltose LEU2RPS1/rps1::CIP30-GPD2*	This study	N/A
CAI4: *ura3::imm434/ura3::imm434 iro1/iro1::imm434*	(Fonzi and Irwin, 1993)	N/A
CAI4-pSM2: *ura3::imm434/ura3::imm434 iro1/iro1::imm434*::pSM2	(Hall et al., 2010)	N/A
CAI4-pSM2-*GPD2*: *ura3::imm434/ura3::imm434 iro1/iro1::imm434*::pSM2-pTef2-*GPD2*	This study	N/A
*ebp1*Δ: As CAI4 but *ebp1*::dlp200/*ebp1*::dlp200	This study	N/A
As CAI4 but *ebp1*::dlp200/*ebp1*::dlp200-pSM2	This study	N/A
CAF2-1: *URA3/ura3:: λimm434*	(Fonzi and Irwin, 1993)	N/A
*cdr1*Δ: As CAF2-1, but *crd1::hisG/cdr1:hisG-URA3-HisG*	(Sanglard et al., 1997)	N/A
*cdr1/2*Δ: As CAF2-1, but *crd1::hisg/cdr1:hisG*, *cdr2::hisG-URA3-hisG/cdr2::hisG*	(Sanglard et al., 1997)	N/A
**Chemicals, peptides, and recombinant proteins**		
Purified C3	Sigma, UK	C2910
Purified C3b	Sigma, UK	204860
Aniline Blue fluorochrome	Bioscience supplies	100-1
Fc-Dectin-1	G. Brown, University of Aberdeen	N/A
TRITC-conjugated wheat germ agglutinin	Molecular Probes, Life Technologies	W849
TRITC-conjugated concanavalin A	Molecular Probes, Life Technologies	C860
Alexa fluor 488 Conjugated concanavalin A	Molecular Probes, Life Technologies	C11252

(*Continued on next page*)

Continued

REAGENT or RESOURCE	SOURCE	IDENTIFIER
Deposited data		
Raw and analysed RNA seq data:	This paper	GSE145240
Experimental models: Cell lines		
J774A.1 mouse macrophage like cell line	Sigma, UK	91051511-1VL
NIH3T3 fibroblast cell line	G. Brown, University of Aberdeen	N/A
NIH3T3 expressing Fc-Dectin-1	G. Brown, University of Aberdeen	N/A
Experimental models: Organisms/strains		
Wild type (AB) *Danio rerio* Zebrafish	University of Birmingham	N/A
Oligonucleotides		
GPD2-SacI-F: CTCCGAGCTCGGTGATGGTGATGGTGATGG	This paper	N/A
GPD2-NotI-R: GGAGAGCGGCCGCTGGTAAATTGGACAACGAGTGG	This paper	N/A
GPD2-OE-F: **GGAGAG**ccgcggATGACTACTTCCCCATATCCAAT	This paper	N/A
GPD2-OE-R: **GGAGAG**gcggccgcACGCAGAGAACAAGAACGTC	This paper	N/A
EBP1-5F: CTCCGATATCATCGCATGAG	This paper	N/A
EBP1-5R: GGAGAGGAGCTCctgatgatatgataatttgc	This paper	N/A
EBP1-3F: GGAGAGAAGCTTGGGAATGAAGTTCTATTAGC	This paper	N/A
EBP1-3R: GGAGAGGGTACCgttacatctactactacagg	This paper	N/A
EBP1-CF: ACAACAACAAGAAGACACGG	This paper	N/A
EBP1-CR: GAATCTATGGTCAAGTAGAAG	This paper	N/A
URA3-F: GCCTCACCAGTAGCACAGCGATTA	This paper	N/A
RT-ACT1-F: CCTACGTGTACTTGTGCAAGGCAA	This paper	N/A
RT-ACT1-R: TAGTTGTGTGCACTGAGCGTCGAA	This paper	N/A
RT-*GDP2*-F: GCCAACGAAGTTGCCAAAGGT	This paper	N/A
RT-*GPD2*-R: AGGCACCAGCAATAGAGGCA	This paper	N/A
BCR1-F:GGAGAGgagctcCCAACCAACATTCCAATTCC	This paper	N/A
BCR1-R: GGAGAGgcggccgcAACCTTTACCTTTGGATTTTTGA	This paper	N/A
RT-EBP1-F: TGCGCCATCAGCAGTTTATTGG	This paper	N/A
RT-EBP1-R: TCCAACAAGTAACCATGAGCACCA	This paper	N/A

RESOURCE AVAILABILITY

Lead contact

Further information and requests for resources and reagents should be directed to and will be fulfilled by the lead contact Dr R Hall (r.a.hall@kent.ac.uk).

Materials availability

New strains and materials made during this study will be made available upon request to the lead contact Dr R Hall (r.a.hall@kent.ac.uk) subject to the completion of a materials transfer agreement.

Data and code availability

- The raw RNA Seq data files are available at the Gene Expression Omnibus (GEO) database (http://www.ncbi.nlm.nih.gov/geo/) at the following accession number GEO: GSE145240.
- This paper does not report original code
- Any additional information required to reanalyze the data reported in this paper is available from the lead contact upon request

EXPERIMENTAL MODEL AND SUBJECT DETAILS

Fungi

Candida strains (Table S6) were routinely maintained on YPD agar (1% yeast extract, 1% peptone, 2% glucose and 2% agar). For broth cultures all strains were cultured in YPD (1% yeast extract, 1% bacto-peptone, 2% glucose) buffered to pH6 with 3.57% HEPES. Oestrogen was diluted in 10% ethanol to a stock concentration of 100 μg/ml and diluted into YPD at the required concentrations, maintaining the final ethanol concentration at 0.3%.

Cell lines

J774A.1 macrophages (Sigma, UK) were maintained in low glucose DMEM supplemented with 5% FBS, 2mM L-glutamine and 2 mM Pen/Strep, 37C, 5% CO_2.

Zebrafish

Zebrafish care and experiment protocols were performed in accordance with the Home Office project license 40/3681 and personal license IE905E215 as per Animal Scientific Procedures Act 1986. Wild type (AB) *Danio rerio* zebrafish used in the study were housed in a recirculating system of gallon tanks at the University of Birmingham Zebrafish Facility. To obtain embryos, 4 male and 5 female fish were transferred into a breeding tank and maintained at 28°C, 14 h light/10 h dark cycle. Embryos were collected the following day, sorted and incubated at 30°C for 24 h in E3 media (5 mM NaCl, 0.17 mM KCl, 0.33 mM $CaCl_2$, 0.33 mM $MgSO_4$, 0.00003% methylene blue, pH 7). Embryos were maintained at a density of 100 per 14.5 cm dish containing 150 mL E3 media supplemented with 0.02 mg/mL Phenylthiourea.

Human macrophages and monocytes

Protocols for human blood collection and isolation of neutrophils and peripheral blood mononuclear cells (PBMCs) were approved by the ethical review board of the School of Biosciences at the University of Birmingham. Blood was collected anonymously and on a voluntary basis after getting written informed consent.

METHOD DETAILS

Phagocytosis experiments

Phagocytosis assay was performed as previously described (Cottier et al., 2019). Briefly, overnight cultures of *C. albicans* were subcultured 1:100 in fresh YPD media, media supplemented with 0.3% ethanol, or media supplemented with oestrogen (0.0001 ⌠M, 0.01 μM or 10 μM) and incubated at 37ºC, 200 rpm for 4 h. *C. albicans* cells were washed three times in PBS and 1 x10^5 J774A.1 macrophages (Sigma, UK) were infected with 5 x 10^5 yeast cells (multiplicity of infection [MOI] of 5) for 45 min at 37°C, 5% CO_2. Cells were washed with PBS to remove non-phagocytosed yeast cells, and phagocytosis stopped by fixing with 4% paraformaldehyde (PFA) for 20 minutes. To distinguish between *C. albicans* cells that are associated with the surface of the innate immune cells, and those that have undergone phagocytosis and are truly internalised, samples were stained for 30 minutes with 50 μg/ml ConA-FITC which is non-permeable and can only bind cells that have not been phagocytosed, washed and imaged. Phagocytosis events were scored from multiple fields of view using imageJ and expressed as the phagocytic index (phagocytosis events/100 macrophages). When required, J774A.1 cells were maintained in DMEM supplemented with heat inactivated serum and the assay was complemented with 1 μg/mL C3 (Sigma, C2910) or C3b (Sigma, 204860).

Human macrophages and neutrophils

PBMCs and neutrophils were isolated as previously described (Sherrington et al., 2017). Neutrophils were seeded at 2 x 10^5 cells/mL in 24-well plates in serum free RPMI supplemented with 100 mM L-glutamine, incubated for 1 h at 37°C, 5% CO_2 and then co-incubated with 1 x 10^6 *C. albicans* cells (MOI = 5) for 45 min at 37°C, 5% CO_2. Cells were immediately fixed with 4% PFA and stained with 50 μg/ml ConA-FITC for 30 minutes to stain non-phagocytosed fungal cells, washed and imaged. Phagocytosis events were scored from multiple fields of view using imageJ. To assess primary macrophage phagocytosis rates PBMCs (0.5 x10^6) were seeded into 24 well plates in differentiation media (RPMI 1640 supplemented with 100 mM L-glutamine, 10% human AB serum, 100 mM Pen/Strep and 20 ng/ml M-CSF) for 7 days replacing the media every 2-3 days, and then phagocytosis rates determined as described above. To assess cytokine production 2.5 x10^4 PBMCs were stimulated with 5 x10^4 PFA fixed *C. albicans* for 24 hours, supernatants collected and stored at -20°C and cytokine concentrations quantified by ELISA.

Genetic manipulation of C. albicans

All primers used in genetic manipulation of *C. albicans* are listed in S2 Table. To reintroduce *GPD2* into *C. albicans gpd2*Δ mutant, the *GPD2* locus was PCR-amplified from *C. albicans* SC5314 genomic DNA using primers GPD2-SacI-F and GPD2-NotI-R. The PCR product was cloned into CIp30 plasmid (Dennison et al., 2005) restricted with *SacI* and *NotI* using T4 DNA ligase. The generated CIp30-*GPD2* was linearized with *StuI* and transformed into *C. albicans gpd2*Δ mutant by standard heat-shock transformation (Walther and Wendland 2003).

To generate a *C. albicans* strain that over expresses *GPD2*, the open reading frame of *GPD2* was cloned into pSM2 (Barkani et al., 2000) using primers GPD2-OE-F and GPD2-OE-R under the control of the *TEF2* promoter. The resulting plasmid was linearized with *PacI*, and integrated into CAI4 at the *URA3* locus by standard heat-shock transformation.

To generate *ebp1*Δ, 500 bp of the 5' and 3' UTR were amplified from genomic DNA using primers EBP1-5F, EBP1-5R, and EBP1-3F and EBP1-3R. The resulting PCR products were purified, digested with *EcoRV* and *SacI* or *HindIII* with *KpnI* and cloned into the mini URA blaster cassette pDDB57 (Wilson et al., 2000). The resulting disruption cassette was digested with *EcoRV* and *KpnI* and transformed in CAI4 by standard heat-shock transformation. Resulting colonies were screened by PCR and positive colonies were plated onto YNB supplemented with 5-fluoroorotic acid (5-FOA) and uridine to select for spontaneous homologous recombination and loss of *URA3*. Resulting colonies were then re-transformed with the *EBP1* knockout cassette, and loss of both alleles was confirmed by PCR using primers EBP1-CF, URA3-F and EBP1-CR, and lack of expression was confirmed by qPCR using primers RT-EBP1-F and RT-EBP1-R. The URA baster cassette was recycled and *URA3* replaced at its native locus by transforming the strain with pSM2.

To complement the *ebp1*Δ, mutant, the *EBP1* ORF together with 800 bp up and downstream were amplified using primers EBP1-F and EBP1-R, and cloned into pSM2 using *SacII* and *NotI* restriction sites. The resulting plasmid with linearized with *PacI* and transformed into *ebp1*Δ, and expression confirmed by RT-PCR.

To overexpress *GPD2* in the *ebp1*Δ mutant pSM2-pTEF2-GPD2 was linearized with PacI and transformed into the *ebp1*Δ *ura3*Δ by standard heat-shock transformation.

To complement the *bcr1*Δ mutant, the *BCR1* ORF together with 900 bp upstream and 500 bp downstream was amplified by PCR using primers BCR1-F and BCR1-R and cloned into CIP30 using *SacI* and *NotI* restriction sites. The resulting plasmid was linearized by *StuI* and transformed into *bcr1*Δ mutant by standard heat-shock transformation.

To overexpress *GPD2* in the *bcr1*Δ mutant, the pTEF2-GPD2 was excised from pSM2-pTEF2-GPD2 and ligated into CIP30 using the *SacI* and *NotI* restriction sites. The resulting plasmid was linearized with *StuI* and transformed into the *bcr1*Δ mutant by standard heat-shock transformation.

Immunofluorescent staining of C. albicans *cell wall components*

C. albicans cells were stained as previously described (Sherrington et al., 2017). Briefly, *C. albicans* cells from overnight culture were sub-cultured in YPD broth with or without oestrogen supplementation and incubated at 37°C, 200 rpm for 4 h. Cells were harvested by centrifugation, washed in PBS and fixed with 4% PFA. To quantify total mannan, glucan and chitin levels in the cell wall, cells were stained with 50 μg/ml TRITC-conjugated concanavalin A (Molecular Probes, Life Technologies), 33.3 μg/ml Aniline Blue fluorochrome (Bioscience supplies) and 3 μg/ml Calcofluor White for 30 minutes. To quantify surface exposure of β1,3-glucan and chitin, cells were stained with 3 μg/ml Fc-Dectin-1 (a gift from G. Brown, University of Aberdeen) and 50 μg/ml TRITC-conjugated wheat germ agglutinin (Molecular Probes, Life Technologies). Fluorescence was quantified on an Attune FACS machine (50 mW Blue/Violet standard configuration), with 10,000 events observed. CFW and Aniline Blue fluorescence intensities were quantified using the 405 nm laser on the Attune in combination with 603/48 and 650DPL filters, FITC labelled cells were quantified using the 488 nm laser in combination with 530/30 and 555DLP filters, and TRITC fluorescence was quantified using the 488 nm laser in combination with 574/26 and 650DLP filters. The MFI was corrected for background fluorescence. FACS data were analysed by Kruskal-Wallis test followed with a post-hoc Dunn's multiple comparisons test at 95% confidence.

RNA sequencing

C. albicans cells were grown for 4 h in YPD broth with or without 10 μM 17β-estradiol at 37ºC, 200 rpm. Cells were harvested by centrifugation, washed three times in PBS, and snapped frozen in liquid nitrogen. Total RNA was extracted as per manufacturer's instructions using the RNeasy Plus mini kit (Qiagen). Total RNA was quantified using a NanoDrop 8000 spectrophotometer (ND-8000-GL; Thermo Fisher). RNA samples were assessed for genomic DNA contamination by PCR and agarose gel electrophoresis. Samples were then processed as previously reported (Cottier et al., 2019). Sequencing reads are available at the Gene Expression Omnibus (GEO) database (http://www.ncbi.nlm.nih.gov/geo/) at the following accession number GEO: GSE145240.

Reads were analysed following a previous published method (Cottier et al., 2019) and using CLC Genomic workbench 11.0.1 software (Qiagen). In summary, adapter and quality trimming was performed before reads were mapped to *C. albicans* reference genome (Assembly 21, version s02-m09-r10). Transcript Per Kilobase Million (TPM) were reported for each open reading frame (ORFs). Statistical analysis was performed after addition to all values of the lowest TPM measurement, then data were log10 transformed and differential expression between conditions were considered significant if the absolute value Fold Change >1.5 and FDR < 0.05. Pathoyeastract GO term finder (Inglis et al., 2012) was used to perform Gene ontology (GO) analysis with P-values corresponding to Bonferroni-corrected hypergeometric test P-values. Motif analyses were performed in the MEME suite website (http://meme-suite.org/).

RT-PCR

C. albicans cells were grown for 4 h in YPD broth with or without 10 μM 17β-estradiol at 37ºC, 200 rpm. Cells were harvested by centrifugation, and snapped frozen in liquid nitrogen. RNA was extracted using the RNeasy Plus mini kit (Qiagen) as per manufacturer's instructions. RNA quality and quantity were checked by electrophoresis and spectroscopy. The qRT-PCR was performed using the 2x qPCRBIO SyGreen mix kit (PCRbiosystem) according to manufacturer's recommendations with 50 ng of total RNA (primers

shown in Table S7). Relative quantification of gene expression was determined by the Delta Delta Ct method with *ACT1* as an endogenous control. mRNA expression was performed in technical triplicate, and data represent the mean and SEM from three independent biological repeats, and were analysed using a paired T-test with 95% confidence.

Complement binding

C. albicans cells were grown for 4 h in YPD broth with or without 10 μM 17β-estradiol at 37ºC, 200 rpm. Cells were harvested, washed three times in PBS, fixed on ice for 30 min with 4% PFA. Then, 2 x 10^6 yeast cells were incubated with 400 μL 10% normal human serum for 20 min at 37ºC, 200 rpm. Cells were washed thrice in PBS and incubated on ice with 100 μL of either 10 μg/mL Anti-Factor H Goat pAb (Sigma, 341,276-1ML) or 1 μg/mL Goat anti Chicken IgY (H+L) diluted in 1% BSA/PBS. Cells were washed thrice in PBS and incubated in dark with 100 μL of either Rabbit anti Goat IgG (H+L) Secondary Antibody, Alexa Fluor 488 (Invitrogen, A11078) or Goat anti Chicken IgY (H+L) Secondary Antibody, Alexa Fluor 594, (Invitrogen, A11042) diluted 1:200 in 1% BSA/PBS. Cells were analysed by flow cytometry and median fluorescence intensity determined. FACS data were analysed by Kruskal-Wallis test followed by a post-hoc Dunn's multiple comparisons test at 95% confidence.

Zebrafish infection

Hind brain infections were performed as previously described (Mallick et al., 2016). Briefly, zebrafish at the prim25 stage were manually dechorionated, and anesthetized in 160 μg/mL Tricaine. Approximately 5 nL of injection buffer (10% PVP-40 in PBS, 0.05% phenol red) or *C. albicans* suspension at 5 × 10^7 cells/mL in injection buffer was microinjected into the hindbrain ventricle via the otic vesicle to achieve a dose of 20-50 yeast/larva. Within 1 h of infection, larvae were screened by microscopy to remove fish noticeably traumatised from microinjection and to ascertain correct injection site and inoculum size. At least 15 larvae per condition were transferred into a 6-well plate, incubated at 28°C in E3 media either with or without 1 μM oestrogen and observed for survival every 24 h until day 5 post fertilisation.

QUANTIFICATION OF STATISTICAL ANALYSIS

Unless indicated otherwise, data were analysed in Prism (version 8) and data presented in graphs represent the mean +/- SEM from at least three independent biological experiments, and the individual biological replicates are displayed on each graph. For phagocytosis data all experiments were performed in technical duplicates, with a minimum of three independent biological repeats. For each technical repeat multiple fields of view were imaged and the mean phagocytosis rate quantified. The mean from each independent biological replicate was then analysed by Kruskal-Wallis test followed by a post-hoc Dunn's multiple comparisons test at 95% confidence. For quantification of immunofluorescence 10,000 cells were analysed by flow cytometry and median fluorescence intensity (MFI) quantified. FACS data were analysed by Kruskal-Wallis test followed by a post-hoc Dunn's multiple comparisons test at 95% confidence. For zebrafish infections, data from three independent biological replicates were pooled together to determine percent survival. Data were analysed by Log-rank Mantel-Cox test and Gehan-Breslow-Wilcoxon test (extra weight for early time points).

Cell Reports, Volume *38*

Supplemental information

Estrogen promotes innate immune evasion of *Candida albicans* through inactivation of the alternative complement system

Pizga Kumwenda, Fabien Cottier, Alexandra C. Hendry, Davey Kneafsey, Ben Keevan, Hannah Gallagher, Hung-Ji Tsai, and Rebecca A. Hall

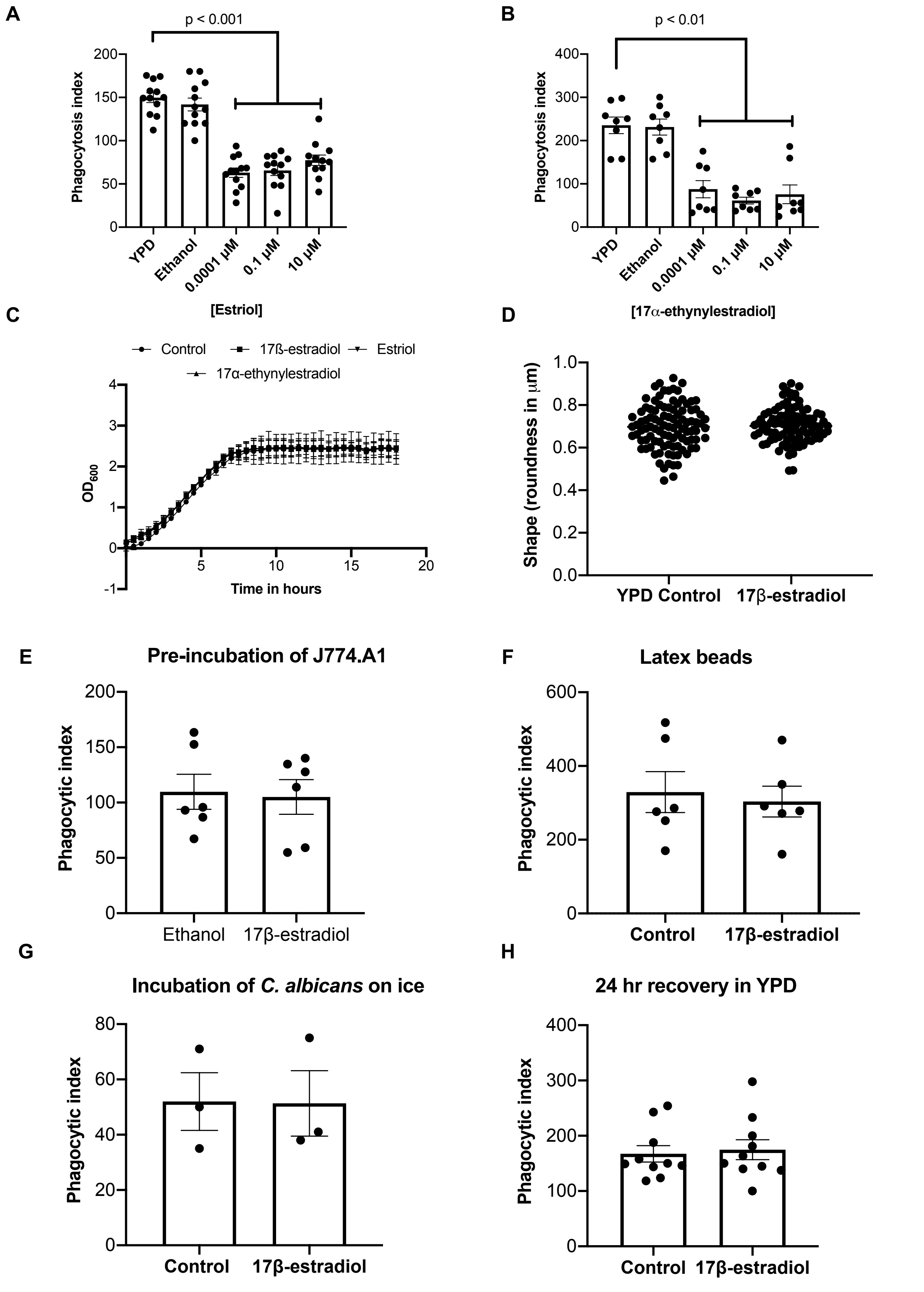

Figure S1. Oestrogen inhibits *C. albicans* phagocytosis, but does not affect fungal growth or morphology related to Figure 1. A) J774A.1 phagocytosis rates of *C. albicans* (SC5314) grown in the presence of estriol.

B) J774A.1 phagocytosis rates of *C. albicans* grown in the presence of 17α-ethynylestradiol. **C)** *C. albicans* cells were grown in 96-welled plate in YPD broth supplemented with 10 μM 17β-estradiol, 10 μM 17α-ethynylestradiol or 10 μM estriol. Optical densities (OD) of the cultures were recorded every 30 min for 18 h. **D)** *C. albicans* was grown in YPD broth supplemented with 17β-estradiol 10 μM for 4 h. Cells were washed with PBS, stained with concanavalin A, imaged by microscopy and analysed for shape. **E)** J774A.1 macrophages preincubated with 10 μM 17β-estradiol were infected with *C. albicans* cells grown in YPD and phagocytosis rates quantified. **F)** Latex beads were incubated at room temperature in PBS with or without 10 μM 17β-estradiol. Beads were washed and co-incubated with J774A.1 macrophages and phagocytosis rates quantified. **G)** *C. albicans* cells were incubated in PBS at 4°C for 4 h with or without 10 μM 17β-estradiol. Cells were washed and co-incubated with J774A.1 macrophages and phagocytosis rates quantified **H)** *C. albicans* cells previously grown in YPD with or without 10 μM 17β-estradiol were harvested, washed and re-incubated in fresh YPD for 24 h. Cells were co-incubated with J774A.1 macrophages and phagocytosis rates quantified. All data represent the mean ± SEM from at least three independent experiments.

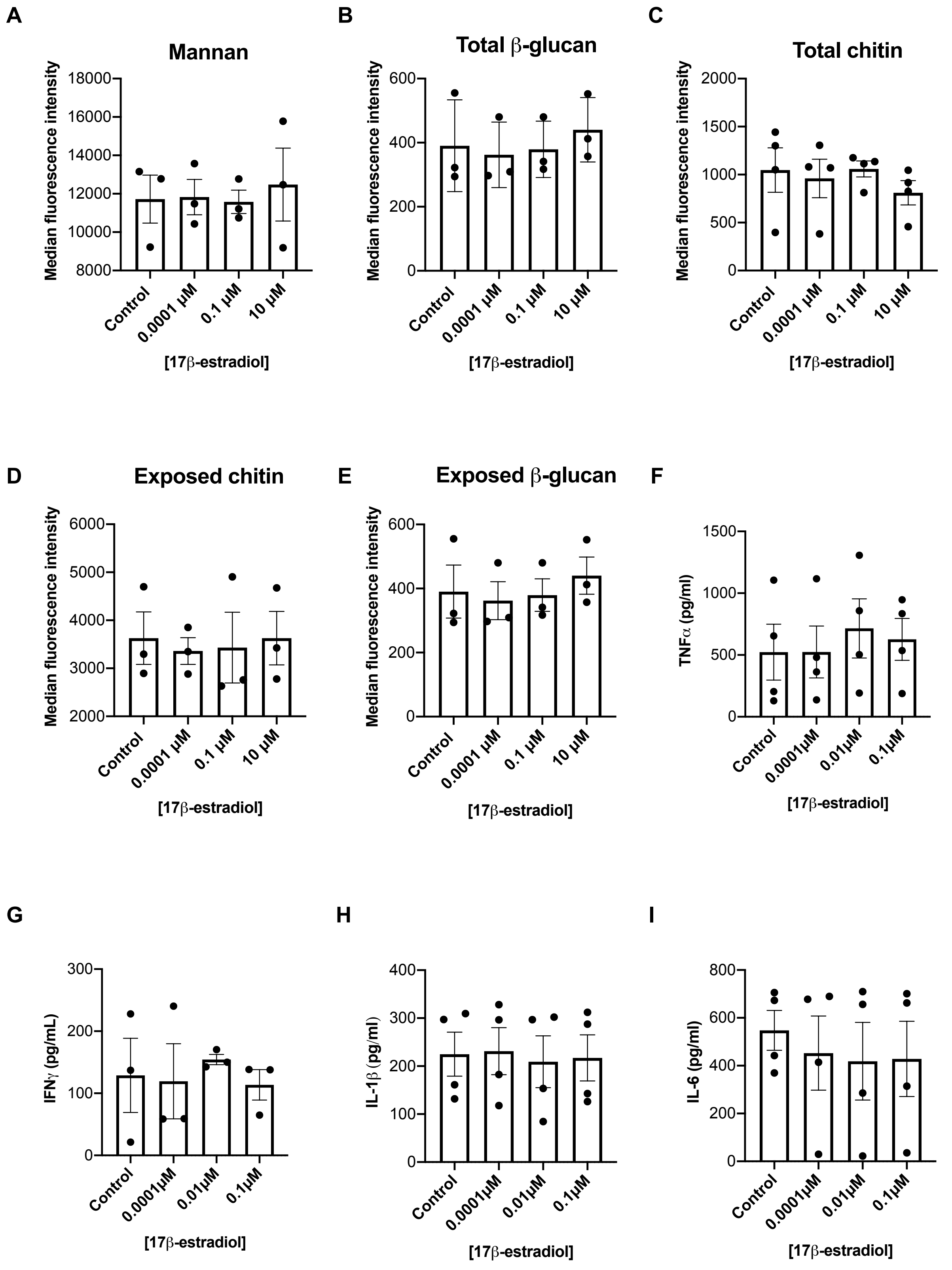

Figure S2. Reduced phagocytosis rates are not correlated with altered cytokine secretion or gross changes in cell wall carbohydrates related to Figure 2. *C. albicans* cells were grown in YPD with or without 10 µM 17β-estradiol. Cells were harvested, washed in PBS, fixed with 4% PFA and stained for **A**) total mannan **B**)

total glucan **C**) total chitin **D)** exposed chitin **E)** exposed glucan. Staining was quantified by flow cytometry and median fluorescence intensities (MFI) determined. All data represent the mean ± SEM from at least three independent biological experiments. *C. albicans* cells were grown in YPD with or without 10 μM 17β-estradiol for 4 h. Cells were washed, fixed with 4% PFA and co-incubated with PBMCs for 24h. Secretion of **F**) TNFα, **G)** IFNγ, **H)** IL-1β and **I)** IL-6 by PBMCs was quantified by ELISA. Data represent the mean ± SEM from four independent experiments using different donors.

Table S2. Gene Ontology analysis of differentially regulated genes (related to Figure 3)

GO Term	Cluster	Adjusted P-value	Genes
**Up regulated genes**			
Hormone binding	2 / 59 genes (3.4%)	0.00473	*CDR1, EBP1*
snoRNA binding	4 / 59 genes (6.8%)	0.00541	*BMS1, NOP14, HCA4, NOP10*
catalytic activity, acting on a rRNA	3 / 59 genes (5.1%)	0.01756	*DIM1, SPB1, CR_04170W_A*
rRNA methyltransferase activity	3 / 59 genes (5.1%)	0.01756	*DIM1, SPB1, CR_04170W_A*
U3 snoRNA binding	3 / 59 genes (5.1%)	0.02147	*BMS1, NOP14, HCA4*
FMN binding	3 / 59 genes (5.1%)	0.0737	*OYE32, EBP1, OYE23*
**Down regulated genes**			
Oxidoreductase activity	19 / 76 genes (25.0%)	2.07E-05	*HMX1, CSH1, IFD6, C1_04460C_A, CAT1, GCV2, C2_00180C_A, ALD5, C2_04480W_A, PST1, PST2, FOX2, POX1-3, MET13, SOU1, GRP2, HPD1, FDH1, FRE7*
Oxidoreductase activity, acting on the CH-OH group of donors, NAD or NADP as acceptor	7 / 76 genes (9.2%)	0.00078	*CSH1, IFD6, FOX2, SOU1, GRP2, HPD1, FDH1*
Oxidoreductase activity, acting on CH-OH group of donors	7 / 76 genes (9.2%)	0.00115	*CSH1, IFD6, FOX2, SOU1, GRP2, HPD1, FDH1*
Aryl-alcohol dehydrogenase (NAD+) activity	2 / 76 genes (2.6%)	0.02754	*CSH1, IFD6*
NAD(P)H dehydrogenase (quinone) activity	2 / 76 genes (2.6%)	0.05466	*PST1, PST2*
Glutaminase activity	2 / 76 genes (2.6%)	0.05466	*SNZ1, SNO1*
Glucose transmembrane transporter activity	3 / 76 genes (3.9%)	0.08953	*HGT19, HGT5, HGT10*

Table S3. Motif letter-probability matrix (related to Figure 3)

Motif	*A*	*C*	*G*	*T*

Motif 1
E-value = 1.2e-159

0.000000	0.037037	0.175926	0.787037
0.009259	0.046296	0.129630	0.814815
0.000000	0.379630	0.000000	0.620370
0.101852	0.074074	0.064815	0.759259
0.000000	0.120370	0.055556	0.824074
0.018519	0.259259	0.009259	0.712963
0.000000	0.259259	0.000000	0.740741
0.055556	0.129630	0.009259	0.805556
0.000000	0.240741	0.009259	0.750000
0.000000	0.157407	0.129630	0.712963
0.083333	0.416667	0.027778	0.472222
0.151389	0.232870	0.001389	0.614352
0.000000	0.185185	0.000000	0.814815
0.055556	0.129630	0.046296	0.768519
0.018519	0.546296	0.120370	0.314815
0.083333	0.037037	0.018519	0.861111
0.037037	0.157407	0.092593	0.712963
0.129630	0.277778	0.046296	0.546296
0.166667	0.314815	0.055556	0.462963
0.129630	0.092593	0.037037	0.740741
0.138889	0.222222	0.083333	0.555556
0.129630	0.157407	0.055556	0.657407
0.037037	0.194444	0.037037	0.731481
0.194444	0.000000	0.111111	0.694444
0.083333	0.333333	0.120370	0.462963
0.203704	0.268519	0.027778	0.500000
0.185185	0.342593	0.046296	0.425926
0.175926	0.194444	0.037037	0.592593
0.111111	0.287037	0.101852	0.500000

Motif 2
E-value = 4.9e-04

0.321429	0.392857	0.071429	0.214286
0.232143	0.214286	0.285714	0.267857
0.357143	0.375000	0.000000	0.267857
0.071429	0.464286	0.000000	0.464286
0.125000	0.428571	0.107143	0.339286
0.107143	0.178571	0.000000	0.714286
0.232143	0.464286	0.017857	0.285714
0.089286	0.392857	0.053571	0.464286
0.178571	0.678571	0.125000	0.017857
0.166964	0.466964	0.002679	0.363393
0.000000	0.821429	0.053571	0.125000
0.232143	0.375000	0.017857	0.375000
0.000000	0.660714	0.000000	0.339286
0.000000	0.732143	0.000000	0.267857
0.196429	0.482143	0.000000	0.321429
0.017857	0.660714	0.089286	0.232143
0.107143	0.589286	0.035714	0.267857
0.000000	0.589286	0.000000	0.410714
0.000000	0.821429	0.000000	0.178571
0.375000	0.089286	0.053571	0.482143
0.392857	0.482143	0.125000	0.000000

Motif 3
E-value = 2.5e-009

0.805556	0.055556	0.138889	0.000000
0.763889	0.097222	0.000000	0.138889
0.500000	0.430556	0.069444	0.000000
0.722222	0.069444	0.111111	0.097222
0.902778	0.013889	0.083333	0.000000
0.652778	0.222222	0.000000	0.125000
0.847222	0.000000	0.152778	0.000000
0.972222	0.027778	0.000000	0.000000
0.847222	0.083333	0.069444	0.000000
0.902778	0.027778	0.000000	0.069444
1.000000	0.000000	0.000000	0.000000
0.791667	0.208333	0.000000	0.000000
1.000000	0.000000	0.000000	0.000000
1.000000	0.000000	0.000000	0.000000

Table S4. Transcription factors that bind identified DNA binding motifs (related to Figure 3)

**Motif**	***S. cerevisiae***	***C. albicans***	**Description on CGD**
**Motif 1**	Azf1	CR_02510W	Induced by Mnl1 under weak acid stress
	Fkh1	Fkh2	Morphogenesis regulator
	Hsf1	Cta8	Mediates heat shock transcriptional induction
	Sfl1	Sfl1	Negative regulation of morphogenesis, flocculation and virulence
	Ste12	Cph1	Mating, and filamentation on solid media repressed
	Cup2	NA	
	Fkh2	NA	
**Motif 2**	Haa1	Cup2	Required for normal resistance to copper
	Msn2	Msn4	Similar to *S. cerevisiae* Msn4
	Msn4	Msn4	Similar to *S. cerevisiae* Msn4
	Ygr067c	Try5	Regulator of yeast form adherence
	Rap1	Rap1	Binds telomeres and regulatory sequences in DNA
	Yml081w	Zms1	Spider biofilm induced
	Cha4	Tea1	Putative transcription factor with zinc cluster DNA-binding motif
	Nrg1	Nrg1	Regulates chlamydospore formation, hyphal gene induction, virulence
	Rgm1	NA	
	Rph1	Rph1	Ortholog(s) have DNA-binding transcription repressor activity
	Gis1	NA	
	Usv1	Bcr1	Regulates a/alpha biofilm formation, matrix, cell-surface-associated genes
	Yer130c	Mnl1	induces transcripts of stress response genes via SLE (STRE-like) elements
	Ypr022c	C3_06150W	Ortholog(s) have role in negative regulation of transcription by RNA polymerase II
**Motif 3**	Azf1	CR_02510W	Induced by Mnl1 under weak acid stress
	Cup2	NA	
	Hcm1	Hcm1	Similar to *S. cerevisiae* Hcm1

	Fkh2	NA	

Table S5. GO Ter analysis of *BCR1* regulated gene (related to Figure 3)

**GO ID**	**GO Term**	**% of input genes**	**p-value**	**Genes**
GO:0009986	cell surface	15.56%	4.91E-07	*ALS4, ECM331, GPD2, PGA13, RBT5, SAP10, SIM1*
GO:0005886	plasma membrane	15.56%	4.61E-05	*AQY1, CFL5, DIP5, ECM331, GPR1, JEN1, RBT5*
GO:0009277	fungal-type cell wall	13.33%	6.29E-08	*ALS4, ECM331, PGA13, RBT5, SAP10, SIM1*
GO:0016021	integral component of membrane	13.33%	9.06E-05	C1_00660C_A, *GPR1, JEN1, MAL31, RBD1, UFE1*
GO:0005634	nucleus	13.33%	1.81E-03	CR_03700C_A, *GRF10, SEF2, SFL2, WOR2, WOR3*

Table S6. Strains used in this study (related to STAR methods)

**Strain**	**Genotype**	**Source/reference**
SC5314	Wild type	(Gillum *et al.* 1984)
SVS006B	Clinical isolate	Prof Ramage, Glasgow University
SVS062A	Clinical isolate	Prof Ramage, Glasgow University
SN152	*arg4Δ/arg4Δ leu2Δ/leu2Δ his1Δ/his1Δ URA3/ura3Δ:: λimm434 IRO1/iro1Δ:: λimm434*	(Noble *et al.* 2010)
SN250	*his1Δ/his1Δ, leu2Δ::C. dubliniensis HIS1 /leu2Δ::C. maltosa LEU2-arg4Δ/arg4Δ, URA3/ura3Δ::imm434-IRO1/iro1Δ::imm434*	(Noble et al. 2010)
SN250-CIP30	As SN250 but *RPS1/rps1::*CIP30	This study
*rob1*Δ	As SN152 but *rob1Δ::C. dubliniensis HIS1/rob1Δ::C. maltose LEU2*	(Noble *et al.* 2010)

*bcr1*Δ	*arg4Δ/arg4Δ leu2Δ/leu2Δ his1Δ/his1Δ URA3/ura3Δ::λimm434 IRO1/iro1Δ:: λimm434 bcr1::LEU/bcr1::HIS1*	(Noble *et al.* 2010)
*bcr1*Δ-CIP30	As *bcr1*Δ but with *RPS1/rps1*::CIP30	This study
*bcr1*Δ-CIP30-*BCR1*	As *bcr1*Δ but with *RPS1/rps1*::CIP30-*BCR1*	This study
*bcr1*Δ-CIP30	As *bcr1*Δ but with *RPS1/rps1*::CIP30-*pTEF2-GPD2*	This study
*gpd2*Δ	As SN152 but *gpd2Δ::C. dubliniensisHIS1/gpd2Δ::C. maltose LEU2*	(Noble *et al.* 2010)
*gpd2*Δ–CIP30	As SN152 but *gpd2Δ::C. dubliniensisHIS1/gpd2Δ::C. maltose LEU2, RPS1/rps1::CIP30*	This study
*gpd2*Δ-CIP30-*GPD2*	As SN152 but *gpd2Δ::C. dubliniensisHIS1/gpd2Δ::C. maltose LEU2RPS1/rps1::CIP30-GPD2*	This study
CAI4	*ura3::imm434/ura3::imm434 iro1/iro1::imm434*	(Fonzi and Irwin 1993)
CAI4-pSM2	*ura3::imm434/ura3::imm434 iro1/iro1::imm434::*pSM2	(Hall *et al.* 2010)
CAI4-pSM2-*GPD2*	*ura3::imm434/ura3::imm434 iro1/iro1::imm434::*pSM2-p*TEF2-GPD2*	This study
*ebp1*Δ	As CAI4 but *ebp1::*dlp200/*ebp1::*dlp200	This study
*ebp1*Δ-pSM2	As CAI4 but *ebp1::*dlp200/*ebp1*::dlp200-pSM2	This study
*ebp1*Δ-pSM2-*EBP1*	As *ebp1*Δ but with pSM2-*EBP1*	This study
*ebp1*Δ-pSM2-*GPD2*	As *ebp1*Δ but *with* pSM2-p*TEF2-GPD2*	This study
CAF2-1	*URA3/ura3:: λimm434*	(Fonzi and Irwin 1993)
*cdr1*Δ	As CAF2-1, but *crd1::hisG/cdr1:hisG-URA3-HisG*	(Sanglard *et al.* 1997)
*cdr1/2*Δ	As CAF2-1, but *crd1::hisg/cdr1:hisG, cdr2::hisG-URA3-hisG/cdr2::hisG*	(Sanglard *et al.* 1997)

Table S7. Primers used in this study (related to STAR methods)

Primer	Sequence
GPD2-SacI-F	CTCCGAGCTCGGTGATGGTGATGGTGATGG
GPD2-NotI-R	GGAGAGCGGCCGCTGGTAAATTGGACAACGAGTGG
GPD2-OE-F	**GGAGAG**ccgcggATGACTACTTCCCCATATCCAAT
GPD2-OE-R	**GGAGAG**gcggccgcACGCAGAGAACAAGAACGTC
EBP1-5F:	CTCCGATATCATCGCATGAG
EBP1-5R:	GGAGAGGAGCTCctgatgatatgataatttgc
EBP1-3F:	GGAGAGAAGCTTGGGAATGAAGTTCTATTAGC
EBP1-3R:	GGAGAGGGTACCgttacatctactactacagg
EBP1-CF	ACAACAACAAGAAGACACGG
EBP1-CR	GAATCTATGGTCAAGTAGAAG
URA3-F	GCCTCACCAGTAGCACAGCGATTA
EBP1-F	GGAGAGccgcggGAAACGCCTTGTTGGACAAA
EBP1-R	GGAGAGgcggccgcTGATAATAACCAGACCACCACA
BCR1-F	GGAGAGgagctcCCAACCAACATTCCAATTCC
BCR1-R	GGAGAGgcggccgcAACCTTTACCTTTGGATTTTTGA
RT-ACT1-F	CCTACGTGTACTTGTGCAAGGCAA
RT-ACT1-R	TAGTTGTGTGCACTGAGCGTCGAA
RT-GPD2-F	GCCAACGAAGTTGCCAAAGGT
RT-GPD2-R	AGGCACCAGCAATAGAGGCA
RT-EBP1-F	TGCGCCATCAGCAGTTTATTGG
RT-EBP1-R	TCCAACAAGTAACCATGAGCACCA
